# Specific mitotic events drive left-right organizer development

**DOI:** 10.1242/dev.204687

**Published:** 2025-05-19

**Authors:** Yan Wu, Yiling Lan, Favour Ononiwu, Abigail Poole, Kirsten Rasmussen, Jonah Da Silva, Abdalla Wael Shamil, Li-En Jao, Heidi Hehnly

**Affiliations:** ^1^ Department of Biology, Syracuse University, Syracuse, NY 13244, USA; ^2^ BioInspired Institute, Syracuse University, Syracuse, NY 13244, USA; ^3^ Department of Biology & Biotechnology, Worcester Polytechnic Institute, Worcester, MA 01609, USA; ^4^ Department of Cell Biology and Human Anatomy, University of California Davis School of Medicine, Sacramento, CA 95817, USA

**Keywords:** Left-right organizer, Cell patterning, Rosette, Lumen formation, Mitosis, Cytokinesis, Microtubules, Cilia

## Abstract

Cell proliferation is crucial for tissue development. Here, we investigate its role in the left-right organizer, which establishes the left-right axis. In zebrafish, we mapped mitotic events in Kupffer's vesicle (KV) and identified an anteriorly enriched, FGF-dependent mitotic pattern. Laser ablation of mitotic cells and pericentrin-null mutants, both reducing mitotic events, resulted in smaller lumens, confirming that cell division is essential for KV development. Pericentrin-null mutants also exhibited defects in leftward cardiac jogging, indicative of KV dysfunction. Using a KV-specific fluorescent microtubule marker, we found that the KV rosette is a transient, centrally organized cluster interconnected by cytokinetic bridges and containing microtubule bundles. This structure emerges after the first four divisions and precedes lumen formation. Mitotic events during KV rounding coincide with rosette formation, spindle rotation and cell extrusion, likely driven by increased packing. Eliminating the first four mitotic events disrupted rosette formation and prevented normal KV rounding. These findings demonstrate that mitotic events are crucial for KV development, with cell division timing shaping KV architecture and function.

## Teaser

**Highlighted Article:** Early, FGF-dependent Kupffer's vesicle progenitor cell divisions are essential for rosette formation, Kupffer's vesicle morphogenesis and proper left-right axis development in zebrafish, showing that mitotic events shape left-right organizer architecture and function.

## INTRODUCTION

The left-right organizer (LRO) is one of the earliest ciliated organs formed during vertebrate development. It is a small and yet important structure – known as the ventral node in mice and Kupffer's vesicle (KV) in zebrafish – that only exists transiently during early embryogenesis to help establish left-right patterning of the body plan. The LRO initiates the development of left-right asymmetry through its cilia-driven, directional fluid flow, which induces asymmetric gene expression (Dasgupta and Amack, 2016; Forrest et al., 2022; Grimes and Burdine, 2017; Okabe et al., 2008). Compromised formation of LRO, such as disruption in lumen formation (Forrest et al., 2022; Navis et al., 2013) or its cells having abnormal ciliary motility (e.g. caused by mutations in the axonemal dynein motor; Layton, 1976; Lee and Anderson, 2008; McGrath et al., 2003) would lead to left-right patterning defects.

We are interested in potential mutations that may reduce the proliferation of LRO progenitor cells and exploring their effects on LRO development and left-right axis determination. Our focus includes the centrosome-associated protein pericentrin. The centrosome is an organelle essential for efficient spindle assembly and function. Variants of or loss of pericentrin (PCNT) have been implicated in microcephalic primordial dwarfism disorders, including Majewski osteodysplastic primordial dwarfism type II (MOPD II). This autosomal recessive disorder is characterized by a range of structural abnormalities, most notably severe microcephaly and short stature. Additional symptoms include aneurysms, insulin resistance, chronic kidney disease, cardiac malformations and systemic vascular disease (Bober and Jackson, 2017). However, direct evidence linking pericentrin loss-of-function mutations to left-right axis defects is limited. Studies reveal that pericentrin is crucial for centrosomal functions, such as spindle orientation and microtubule anchoring, which affect cell proliferation and tissue organization (Chen et al., 2014). We hypothesized that loss of PCNT may disrupt LRO development, causing defects in left-right axis development.

Previous studies using mouse and zebrafish models (for reviews of studies in fish, see Dasgupta and Amack, 2016; Grimes and Burdine, 2017; for a study in mice, see Gredler and Zallen, 2023) have shown that LRO is formed through the organization of the LRO progenitor cells into rosette-like structures before displaying epithelial-like char­acteristics and initiating lumen formation. Lumen formation in *in vitro* 3D cultures of Madin–Darby canine kidney (MDCK) cells suspended in extracellular matrix has been shown to be preceded by cell division, followed by the establishment of apical polarity and cell–cell contacts adjacent to the cytokinetic bridge (Blasky et al., 2015; Klinkert et al., 2016; Li et al., 2014; Mangan et al., 2016). However, these 3D cell culture models do not recapitulate the mesenchymal-to-epithelial transition, as the cells are already epithelial, and they fail to capture the dynamic changes in cell shape and expression profiles observed during left-right organizer formation *in vivo*.

To understand how a functional LRO is formed from a small group of precursor cells to a fluid-filled sac lined with ciliated cells (Aljiboury et al., 2023; Tavares et al., 2017), we examine here the development of the KV at single-cell resolution. Our previous work (Krishnan et al., 2022; Rathbun et al., 2020a) and others’ studies (Abdel-Razek et al., 2023) have provided compelling evidence that mitotic events are present and likely play an important role during KV development. Our findings herein reveal that early mitotic events in the anterior KV, occurring when it comprises fewer than 20 progenitors, are crucial for proper KV formation, whereas later divisions are dispensable.

## RESULTS

### KV developmental defects and loss of mitotic events in pericentrin-null embryos are associated with reduced survival and increased left-right axis defects

**Fig. 1.**
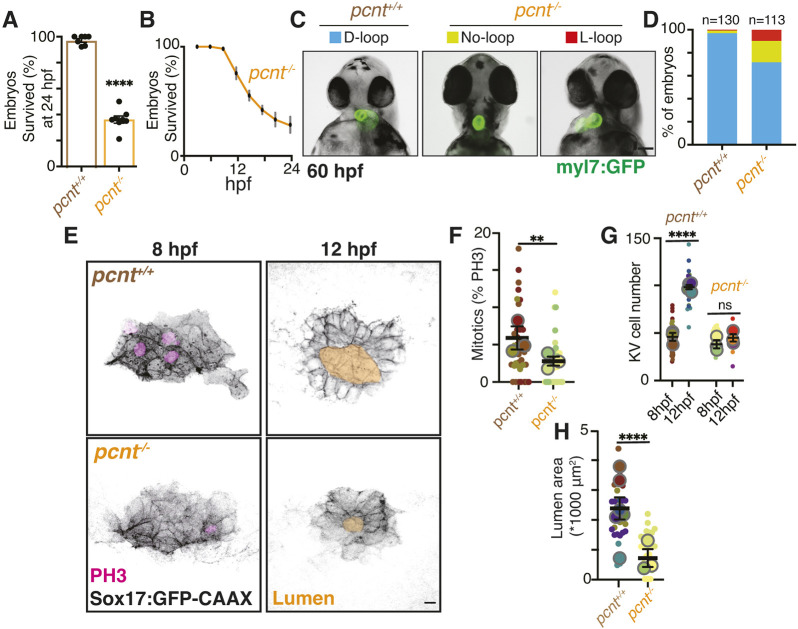
**KV developmental defects and loss of mitotic events in pericentrin-null embryos are associated with reduced survival and increased left-right axis defects.** (A) Percentage embryo survival from wild-type control crosses and embryos from pericentrin-null (*pcnt*^−/−^) crosses. *n*≥7 clutches shown at 24 hpf; each dot represents one clutch. Data are mean±s.e.m. ****P<0.0001 (unpaired, two-tailed Student's *t*-test). (B) Percentage *pcnt*^−/−^ embryo survival from 1 hpf to 24 hpf across three clutches. Error bars represent s.e.m. (C) Representative images of 60 hpf wild-type and *pcnt*^−/−^ fish with Tg(myl7:GFP) hearts shown with a normal cardiac loop (D-loop), an inverted loop (L-loop) and no loop. Scale bar: 100 μm. (D) Percentage of embryos with D-loop, L-loop and no loop calculated from wild-type and *pcnt*^−/−^ clutches combined. *n*=2 clutches measured for wild type, *n*=4 clutches for *pcnt*^−/−^ embryos; total embryo number ≥113 per condition. (E) Confocal projections of pre-lumen (8 hpf, left) and post-lumen (12 hpf, right) KVs lacking pericentrin (*pcnt*^−/−^) compared with wild-type control (*pcnt*^+/+^). KV cell membranes are marked with Sox17:GFP-CAAX (inverted gray), mitotic events with PH3 (magenta). Lumens were traced and highlighted in gold. Scale bar: 10 μm. (F-H) Percentage of mitotic KV cells (F), KV cell number (G) and lumen area (H) were measured for *n*≥23 embryos (small dots represent embryos color coded to match clutch) across *n*≥3 clutches (large dots represent clutch) per condition. Graphs are super plots (as defined by Lord et al., 2020) with mean of clutches shown ±s.e.m. **P<0.01, ****P<0.0001 (unpaired, two-tailed Student's *t*-test); ns, not significant.

Building on prior work establishing mitosis during KV development (Abdel-Razek et al., 2023; Krishnan et al., 2022; Rathbun et al., 2020a), we used maternal-zygotic pericentrin mutants (*pcnt*^−/−^; Sepulveda et al., 2018) to genetically reduce mitotic activity and assess its role in KV formation. Pericentrin is a centrosomal protein known to regulate spindle assembly, and loss of pericentrin can result in a decrease in mitotic entry and failure to complete mitosis (Chen et al., 2014; Delaval and Doxsey, 2010; Mikule et al., 2006; Purohit et al., 1999; Zimmerman et al., 2004). To assess the impact of pericentrin (*pcnt*) loss on early development, we compared the survival rates of *pcnt*^−/−^ embryos to wild-type controls at 24 h post-fertilization (hpf). In *pcnt*^−/−^ embryos, survival rates were significantly reduced, with only 36% surviving compared to 96% in wild-type controls (Fig. 1A). Notably, 25% of *pcnt*^−/−^ embryos succumbed within the first 12 h after fertilization (Fig. 1B). Zebrafish embryo mortality at 12 hpf likely reflects disruptions in early developmental processes, such as gastrulation, axis specification or organogenesis, due to impaired signaling, cytoskeletal dynamics or cellular stress (Bruce and Heisenberg, 2020; Chen et al., 2020; Cooper and Scholpp, 2024; Oteíza et al., 2008; Schauer and Heisenberg, 2021). This is also the developmental window around the time when the left-right axis is being established by the KV (Matsui and Bessho, 2012).

Proper KV function is essential for heart development, as complete loss of KV activity would be expected to randomize heart looping, resulting in approximately equal proportions of normal and abnormal cases. In our analysis of surviving embryos at 60 hpf, we observed left-right axis defects, including abnormalities in heart looping (Fig. 1C). Specifically, while only 3% of wild-type embryos exhibited heart-looping defects, this proportion increased markedly to 28% in *pcnt^−/−^* embryos (Fig. 1D). However, since the KV is not entirely absent but retains a lumen, albeit significantly reduced, it may still provide sufficient function to support normal heart development in 72% of surviving embryos, although this remains significantly below control levels. Together, our findings demonstrate that *pcnt^−/−^* embryos reveal a marked reduction in viability (Fig. 1A,B) and demonstrate heart developmental defects in surviving progeny (Fig. 1C,D).

To determine whether mitotic events were disrupted in the developing KV of surviving *pcnt*^−/−^ embryos compared to controls, we assessed mitotic entry by immunostaining embryos with the mitotic marker phosphorylated histone 3 (PH3). At least 15 embryos per condition were fixed and analyzed at 8 and 12 hpf, using samples from a minimum of three independent clutches. This approach allowed us to evaluate potential reductions in mitotic activity in *pcnt*^−/−^ embryos (Fig. 1E,F). At 8 hpf, KV progenitor cells have not yet formed a lumen, and the developing KV consists of approximately 15-50 cells. By 12 hpf, the KV has expanded to around 100 ciliated cells arranged around a fluid-filled lumen. We found that overall cell division (percentage of PH3-positive cells) of KV precursors decreased significantly in maternal-zygotic *pcnt*^−/−^ embryos compared with control at 8 hpf (Fig. 1E,F). When counting KV cell number at 8 and 12 hpf, we found that both control and mutant conditions had between 15 and 50 KV progenitor cells at 8 hpf, as expected. In controls, this increased to around 100 KV cells by 12 hpf, whereas in mutant conditions it remained at around 50 (Fig. 1G). When measuring lumen area at 12 hpf, *pcnt^−/−^* embryos displayed significantly smaller KV lumens compared to controls, with some embryos failing to form a lumen altogether (Fig. 1E,H). This finding suggests that the reduced number of division events and fewer cells by 12 hpf likely contribute to the significant decrease in lumen area observed in *pcnt^−/−^* embryos.

### KV-specific mitotic events contribute to KV development

**Fig. 2.**
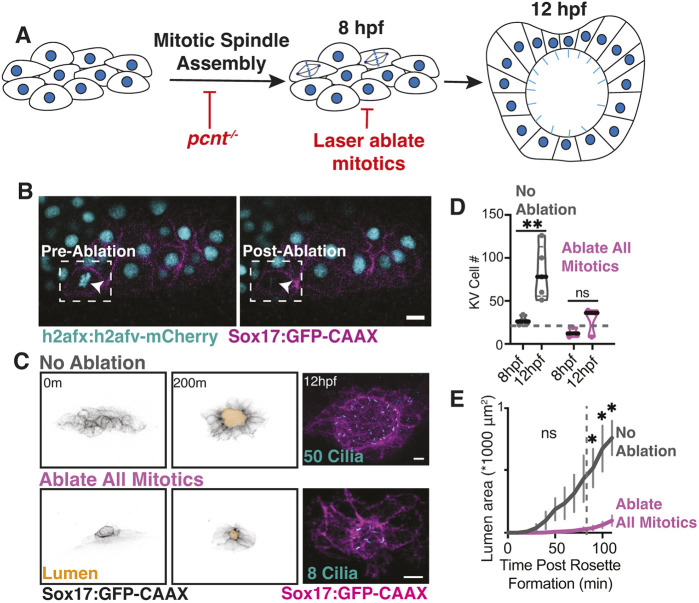
**KV-specific mitotic events contribute to KV development.** (A) Model illustrating experimental approaches to identify the role of mitotic events on KV development. Pale blue lines represent cilia. (B) Representative images of the KV ablation experiment showing a pre-ablation and post-ablation cell. White arrowheads highlight the KV cell ablated. KV cell membranes marked by Sox17:GFP-CAAX (magenta) and nuclei with h2afx:h2afv-mCherry (cyan). Scale bar: 10 μm. (C) Confocal projections taken from a movie acquisition (0-200 min, Sox17:GFP-CAAX, inverted gray; see also Movie 1). Lumens were traced and highlighted in gold. The embryo was fixed at 12 hpf and stained for cilia (acetylated tubulin, cyan; Sox17:GFP-CAAX, magenta). The number of cilia in each representative image is noted. m, min. Scale bars: 10 μm. (D) Truncated violin plot depicting the number of KV cells at 8 hpf and 12 hpf for ‘no ablation’ and ‘all mitotics ablated’ conditions across *n*≥3 embryos (each point represents a single embryo). Median denoted with black line and 20 KV cell threshold demonstrated with gray dashed line. **P<0.01 (unpaired, two-tailed Student's *t*-test); ns, not significant. (E) Lumen area was measured over time for over 100 min for ‘no ablation’ (control) and ‘all mitotics ablated’ conditions. *n*≥3 embryos measured per condition. Error bars represent s.e.m. *P<0.05 (unpaired, two-tailed Student's *t*-test); ns, not significant.

The reduced mitotic activity in *pcnt*^−/−^ embryos prompted us to test directly whether mitosis is required for KV development. To distinguish direct effects from the broader consequences of global *pcnt* loss, we used laser ablation to selectively eliminate mitotic cells within the KV and compared these embryos to non-ablated controls (Fig. 2A,B). To follow division events in KV-specific progenitor cells, a *Tg(Sox17:GFP-CAAX; h2afx:h2afv-mCherry)* line was used, in which the KV progenitor cells were labeled by membrane-bound GFP and chromosomes were labeled with H2afv attached to a red fluorescent protein, mCherry (Fig. 2B). During movie acquisition, mitotic cells were ablated by positioning a region of interest (ROI) over the metaphase plate, distinguished by h2afv-mCherry, and delivering a 355 nm pulsed laser within the ROI (Fig. 2B). Monitoring encompassed all cells, including the cell with the ablated plate, and revealed no evident apoptotic events in the non-ablated cells. The ablated cells failed to complete mitosis and were ultimately extruded from the developing KV.

With these experiments, we imaged a KV within an embryo from 8 hpf to 12 hpf and were careful to start with KVs that contained 20 or fewer KV progenitor cells. When all mitotic events were ablated starting at fewer than 20 KV progenitor cells, the KV was not able to significantly increase in cell number compared to its non-ablation controls (Fig. 2C,D). This finding suggests the importance of mitotic events in KV cell number expansion during development, similar to our observations when comparing *pcnt*^−/−^ embryos to wild-type controls (Fig. 1F). We proceeded to measure lumen area over time and identified significant defects in lumen formation kinetics under conditions in which all mitotic events were ablated compared to the ‘no ablation’ controls (Fig. 2C,E; Movie 1). The same embryos subjected to ablation were fixed at 12 hpf and immunostained for cilia (Fig. 2C). As with lumen formation defects, we found an overall reduction in the number of cilia when mitotic events were ablated (50 cilia under control conditions versus eight cilia under ablated conditions; Fig. 2C). These findings underscore the pivotal role of mitotic events in orchestrating KV lumen formation.

### Identification of an increased number of anteriorly enriched mitotic cells in the KV prior to lumen formation compared to evenly distributed post-mitotic cells after lumen formation

**Fig. 3.**
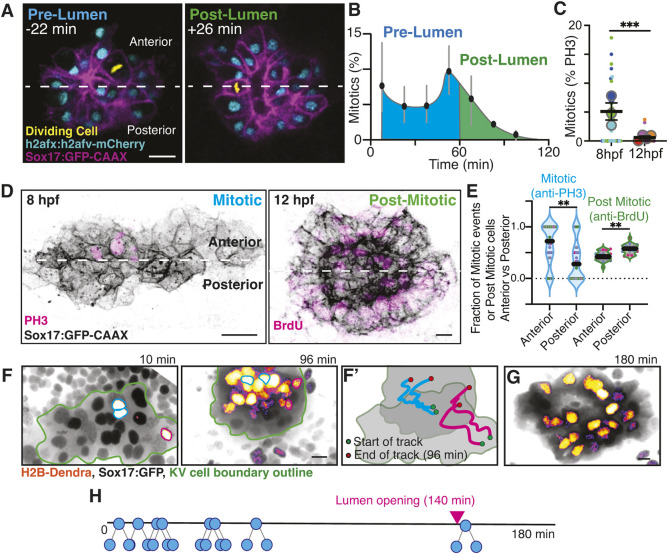
**Identification of an increased number of anteriorly enriched mitotic cells in the KV prior to lumen formation compared to evenly distributed post-mitotic cells post lumen formation.** (A) Representative confocal projections from KV development movie (Movie 2). Pre-KV lumen time point (−22 min before lumen opening) and post-lumen (26 min after lumen opening) are shown. KV cell membranes are marked by Sox17:GFP-CAAX (magenta) and nuclei with h2afx:h2afv-mCherry (non-mitotic, cyan; mitotic, yellow). Scale bar: 10 μm. (B) Percentage of KV cells that are mitotic in 20 min bins (data points) throughout a 120 min movie time course (*n*=3 embryos; error bars represent s.e.m.). (C) Percentage of mitotic KV cells was measured for *n*≥23 embryos (small dots represent embryos color coded to match clutch) across *n*≥3 clutches (large dots represent clutch) at 8 hpf and 12 hpf. Graphs are super plots with mean of clutches ±s.e.m. ***P<0.001 (unpaired, two-tailed Student's *t*-test). (D) Confocal projections of mitotic (PH3, magenta) and post-mitotic (BrdU, magenta) events at pre lumen (8 hpf) and post lumen (12 hpf) stages. KV cell membranes are marked with Sox17:GFP-CAAX (inverted gray). Dashed line indicates division of the KV into anterior and posterior regions. Scale bars: 10 μm. (E) Fraction of PH3- and BrdU-positive cells in the anterior versus posterior regions of the KV (*n*=4 clutches, 36 embryos; and *n*=3 clutches, *n*=17 embryos, respectively, for PH3 and BrdU). Each point represents an embryo, and the point colors represent clutch. **P<0.01 (unpaired, two-tailed Student's *t*-test). (F,G) Confocal projections of H2B-Dendra photoconversion (non-photoconverted black nuclei; photo-converted in fire-LUT). Cyan and magenta outlines mark mitotic photoconverted event at 10 min. A total of eight mitotic events were photoconverted. Daughter cell positioning at 96 min and 180 min is shown (G). (F′) Trajectory of marked mitotic events from division completion to 96 min. KV cell boundary, green outline. Scale bars: 10 μm. (H) Temporal schematic of KV mitotic event for the representative embryo shown in F; the daughter cells did not divide again.

Given the importance of mitotic events in KV lumen formation, we aimed to characterize more precisely their timing in the hour before and after lumen formation. We imaged three *Tg(Sox17:GFP-CAAX; h2afx:h2afv-mCherry)* embryos (example in Movie 2) and synchronized the movies to when the lumen first starts to open. We oriented the KV with the anterior half of the KV towards the top of the embryo and the posterior half of the KV towards the bottom of the embryo. Mitotic events were marked and metaphase plates colored in yellow using Imaris software (Fig. 3A), and compared to non-dividing cells (nuclei; cyan). We found that there was an increased frequency of mitotic events within the hour before the lumen opens (−22 min in Fig. 3A; blue in Fig. 3B) compared to after the lumen opens (+26 min in Fig. 3A; green in Fig. 3B; Movie 2). To characterize this further, we fixed *Tg(Sox17:GFP-CAAX; h2afx:h2afv-mCherry)* embryos at 8 hpf (before lumens form) and 12 hpf (after lumens form) and immunostained for PH3. Across three clutches, we identified that, on average, 5% of KV progenitor cells were undergoing division in a fixed embryo at 8 hpf; in contrast, mitotic events were rare at 12 hpf (Fig. 3C).

While we found an increased incidence of mitotic events before lumen formation compared to after, we also noticed that the mitotic events that occurred were predominantly in the anterior half of the developing KV (Fig. 3A, left, −22 min). To follow up on this observation across a larger population of embryos, we used *Sox17:GFP-CAAX* embryos fixed at 8 hpf and immunostained for PH3 (Fig. 3D, left). We calculated the fraction of mitotic events that occurred in the anterior versus the posterior KV region across four clutches (Fig. 3E, left) and found that there was a significantly higher fraction of mitotic events in the anterior half compared to the posterior (Fig. 3E). Based on this, we hypothesized that post-mitotic daughter cells may be organized more anteriorly in a developed KV with a fully formed lumen at 12 hpf. To test this hypothesis, we incubated embryos with bromodeoxyuridine (BrdU) from 70% epiboly to 12 hpf to label cells that had undergone cell division and identify the post-mitotic cell population. To our surprise, we found that BrdU-positive (post-mitotic) cells were distributed throughout the KV (Fig. 3D, right) with no enrichment to the anterior region (Fig. 3E, right). Based on this finding, we wanted to determine specifically how KV progenitor cells and their progeny reposition following cell division. To do this, we performed cell lineage tracing analysis of H2B-Dendra-expressing, Sox17:GFP-marked KV precursor cells (Fig. 3F). In this experiment, individual H2B-Dendra-positive cells (gray) were photoconverted at metaphase and the trajectories of the resulting daughter cells (red, pseudocolored in a FIRE LUT) were followed throughout KV developmental stages until lumen expansion (Fig. 3F). We found that the daughter cells of a given KV precursor stay together and move within the KV boundary after division (representative events shown in Fig. 3F′). Among eight photoconverted metaphase cells and their progeny that were followed, the daughter cells did not divide again during KV development (depicted along a timeline in Fig. 3H). We found that most of the post-mitotic cells were organized in the anterior region of the KV until just before a lumen was about to form at the 140 min time point (Fig. 3F-H), and then once the lumen started to form, a proportion of the daughter cells were redistributed to the posterior region (Fig. 3G, 180 min). Together, these results indicate that KV progenitor cells divide preferentially in the anterior region of the future KV, and the resulting post-mitotic cells are redistributed throughout the mature KV.

### FGF contributes to mitotic events being enriched at the anterior half of the KV

**Fig. 4.**
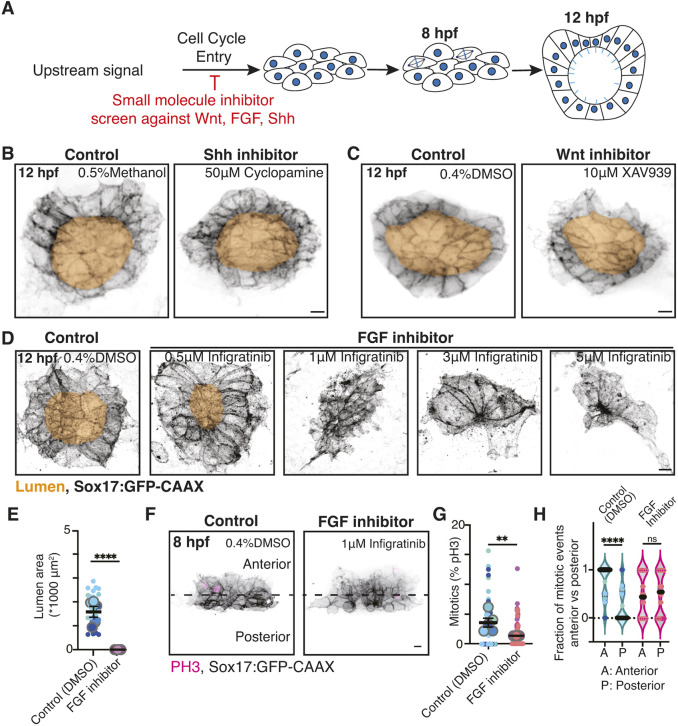
**FGF contributes to mitotic events being enriched at the anterior half of the KV.** (A) Model illustrating the experimental approach to identify whether a signal influences mitotic events during KV development. Pale blue lines represent cilia. (B-D) Confocal projections of post lumen (12 hpf) embryos treated with methanol (control), 50 μM cyclopamine (B), DMSO (control), 10 μM XAV939 (C), or 0.5 μM, 1 μM, 3 μM or 5 μM infigratinib (D). KV cell membranes are marked with Sox17:GFP-CAAX (inverted gray), lumens are highlighted in gold. Scale bars: 10 μm. (E) Lumen area was measured for *n*≥29 embryos per control and 1 μM infigratinib (FGF inhibition) conditions. Super plot is shown with small dots representing each embryo and color coded to match clutch. *n*≥3 clutches were measured with large dots representing clutch. Mean of clutches is shown ±s.e.m. ****P<0.0001 (unpaired, two-tailed Student's *t*-test). (F) Confocal projections of 8 hpf KVs treated with DMSO (control) or 1 μM infigratinib (FGF inhibition). Mitotic events were marked with PH3 (magenta). KV cell membranes were marked with Sox17:GFP-CAAX (inverted gray). Scale bar: 10 μm. (G) Percentage mitotics within developing KVs at 8 hpf was measured for *n*≥53 embryos per control and 1μM infigratinib (FGF inhibition) conditions. Super plot shown with small dots representing each embryo and are color coded to match clutch. *n*≥3 clutches were measured with large dots representing clutch. Mean of clutches shown ±s.e.m. **P<0.01 (unpaired, two-tailed Student's *t*-test). (H) Fraction of PH3-positive cells in the anterior versus posterior regions of the KV at 8 hpf. *n*≥33 embryos were used per control and 1 μM infigratinib (FGF inhibition) conditions. Each point represents an embryo, the point colors represent clutch, *n*≥3 clutches. ****P<0.0001 (unpaired, two-tailed Student's *t*-test); ns, not significant.

Since early mitotic events occurred in the anterior half of the developing KV (Fig. 3), we hypothesized that an anteroposterior signaling mechanism coordinates the anterior enrichment of mitotic events and facilitates subsequent LRO development. To test this, we individually inhibited the major signaling pathways that have been implicated in LRO development, such as fibroblast growth factor (FGF), Wnt and Sonic hedgehog (Shh) (Arrington et al., 2013; Caron et al., 2012; Meyers and Martin, 1999; Molina et al., 2007; Neugebauer et al., 2009), using well-established pharmacological inhibitors, and then determined whether KV development was affected (Fig. 4A). The FGF, Wnt and Shh signaling pathways were inhibited using infigratinib, XAV939 and cyclopamine, respectively, at concentrations within the ranges previously reported in the literature (Arrington et al., 2013; Caron et al., 2012; Meyers and Martin, 1999; Neugebauer et al., 2009). We found that blocking FGF signaling, but not Wnt or Shh signaling, caused significant defects to lumen formation (Fig. 4B-D). Titration of the FGF inhibitor infigratinib revealed significant lumen formation defects at concentrations as low as 1 µM (Fig. 4D). When assessing the severity of these defects across three clutches, we found that none of the embryos formed a lumen at 1 µM infigratinib (Fig. 4E). We then tested whether this concentration of infigratinib affected mitotic entry in KV precursors by fixing FGF-inhibited and vehicle-control embryos at 8 hpf, immunostaining for PH3 (Fig. 4F), and calculating both the mitotic index (Fig. 4G) and the distribution of mitotic events between the anterior and posterior regions (Fig. 4H). We found that FGF inhibition led to a significant decrease in mitotic events (Fig. 4G). We calculated the fraction of mitotic events that occurred in the anterior versus the posterior KV region per embryo across more than three clutches for vehicle control and FGF-inhibited conditions (Fig. 4H). The mitotic events that did occur in FGF-inhibited embryos were not enriched in the anterior region compared to controls (Fig. 4H). These findings suggest that FGF acts as a signal to induce anterior divisions during KV development.

### Early KV divisions stably position spindles until the KV starts rounding, then the few cells that enter mitosis are unable to stably position their spindle and are extruded

**Fig. 5.**
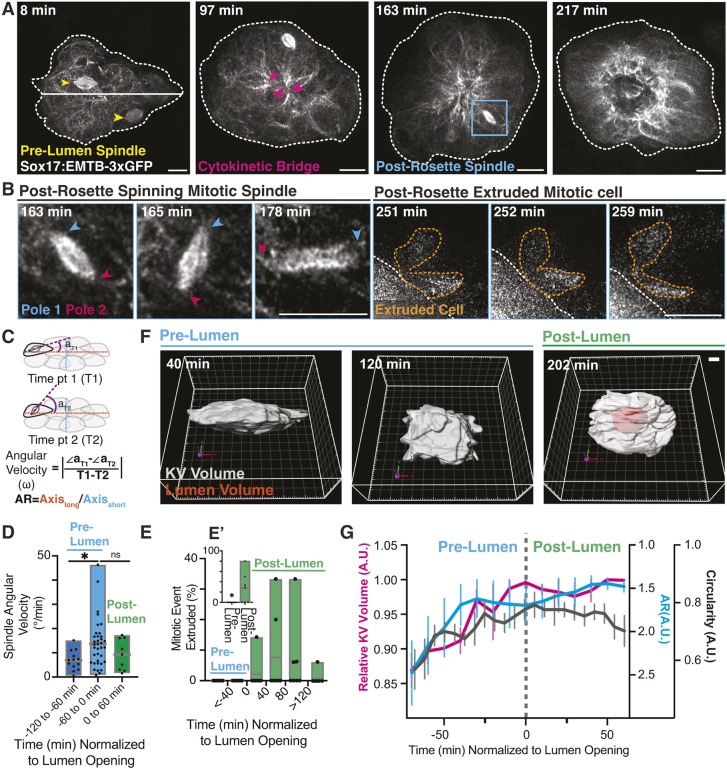
**Early KV divisions stably position spindles until the KV starts rounding, then the few cells that enter mitosis are unable to stably position their spindle and are extruded.** (A,B) Stills from time-lapse movie of microtubule organization during KV formation (Sox17:EMTB-3xGFP, gray). KVs are outlined (white dashed line). Highlighted are spindles (yellow arrowheads) and cytokinetic bridges (pink arrowheads). The blue boxed region is magnified in B showcasing a spinning mitotic spindle that becomes extruded. Mitotic spindle poles are noted with blue and pink arrowheads. An extruded cytokinetic KV cell post-spinning event is outlined (orange dashed line). Scale bars: 10 μm. See also Movie 3. (C) Method of measurement of the KV aspect ratio (AR) and the angular velocity of the spindle (ω∠a), illustrated schematically. (D,E) Floating bar graphs depicting the maximum, minimum and mean of KV spindle angular velocity (D) and mitotic extrusion events (E). For D, 58 spindle poles from seven embryos across six clutches were measured. *P<0.05 (one-way ANOVA). For E, the percentage of extruded mitotic events in 40 min bins relative to lumen formation were measured for seven embryos from six clutches. (E′) The percentage of mitotic events with post mitotic cell extrusions pre- versus post-lumen formation. *n*=7 embryos from 6 clutches. *P<0.05 (unpaired two-tailed *t*-tests). ns, not significant. (F) 3D surface rendering of a representative KV (gray) at pre-lumen stage (40 min, left; 120 min, middle) and post-lumen stage (202 min, right). The *x*-axis, *y*-axis and *z*-axis are indicated by red, green and blue arrows of the reference frame, respectively. Scale bar: 10 μm. (G) Relative KV volume (magenta; *n*=3 from 3 clutches), AR (cyan; *n*=7 from 6 clutches) and circularity (gray; *n*=6 from 6 clutches) over time normalized to lumen opening (marked by gray dashed line). Error bars represent s.e.m. A.U., arbitrary units.

Proper spindle dynamics and positioning during mitosis is important for tissue morphogenesis (Bergstralh et al., 2017; Vertii et al., 2018). Given the significance of early mitotic events in KV development (Figs 1-3), we aimed to understand the relationship between spindle positioning and division timing by analyzing the spindle behaviors in KV cells over time. To label microtubules in KV cells specifically, we adapted a similar strategy we have used before (Rathbun et al., 2020b) to generate a *Tg(Sox17:EMTB-3xGFP)* transgenic line, in which the microtubules in KV cells are labeled by GFP tagging the microtubule-binding domain of the protein ensconsin (Fig. 5A). Through analysis of a series of time-lapse images, we found that the dividing KV precursors stably positioned their spindles around 8 hpf when the KV has a more elongated shape early on in its development (Fig. 5A, 8 min; Movie 3). These stably positioned spindles are defined as those that exhibit minimal wobbling or rotational behavior. As the KV transitions from an elongated to a rounded structure, cells began to organize into a rosette-like arrangement. During this process, the early dividing cells and their associated spindles resolved into cytokinetic cells, with their cytokinetic bridge-associated microtubule bundles oriented toward the rosette center (Fig. 5A, 97 min; Movie 3) where the lumen will eventually form (Fig. 5A, 217 min; Movie 3). This work complements our previous studies in which we found the cytokinetic bridge/midbody markers MKLP1 and RacGAP at KV rosette centers (Rathbun et al., 2020a), but were unable to assess spindle dynamics at the time. As the KV transitioned from an oblong structure with distinct long and short axes (Fig. 5A, 8 min) to a more rounded form (Fig. 5A, 97-163 min), metaphase cells observed at 97 min began to rotate their spindles, after which they were extruded from the KV (Fig. 5A, 163 min; Fig. 5B; Movie 3). This was a novel serendipitous observation made possible by microtubule labeling.

Given the observed increase in spindle rotation that correlated with changes in KV shape, we quantified spindle rotation relative to lumen opening by calculating spindle angular velocity across seven developing KVs (Fig. 5C). Angular velocity measurements were taken during three time intervals: 120-60 min prior to lumen opening, 60 min prior to lumen opening, and 60 min following lumen opening. Between 120 and 60 min before lumen opening, spindle angular velocity averaged below 6.8°/min. This significantly increased to 13.64°/min between 60 and 0 min before lumen formation, with maximum values reaching 46.4°/min (Fig. 5D), before decreasing to 9.19°/min in the 60 min following lumen opening. Notably, a population of these mitotic cells was extruded from the KV as the lumen began to form (Fig. 5E). When combining time points before and after lumen formation, we found a significantly higher number of extrusion events in the post-lumen period (Fig. 5E′).

To investigate whether this mitotic cell extrusion could be driven by changes in cell packing, we measured KV shape and volume dynamics across three to seven embryos by calculating the KV aspect ratio (AR), the cell-occupied volume of the KV using Imaris software (representative images in Fig. 5F), and KV circularity over time. The AR was calculated as the ratio of the longest KV axis to the shortest axis. Time was normalized so that negative values represent pre-lumen formation and positive values represent post-lumen formation. The dashed line in Fig. 5G indicates the time of lumen opening (0 min). Our analysis revealed a consistent trend across all measurements: KV cell volume, circularity and AR increased until lumen opening, after which these metrics stabilized (Fig. 5G). This stabilization coincided with the onset of mitotic cell extrusion from the KV. These findings suggest that as the KV rounds and cell packing increases, mitotic cells entering division may be unable to integrate into the developing KV, potentially due to mechanical constraints imposed by increased cellular density.

### Later cell division events are extruded due to KV rounding and cell packing

To our knowledge, this is the first evidence demonstrating that cell extrusion is an integral part of the program for KV development. This process is reminiscent of cell extrusion observed in other developmental contexts, such as the generation and maintenance of tight epithelial barriers (Anton et al., 2018; Eisenhoffer and Rosenblatt, 2013; Gudipaty and Rosenblatt, 2017; Marinari et al., 2012; Nanavati et al., 2020; Tada, 2021), suggesting that this could be a process that is conserved across all LROs.

**Fig. 6.**
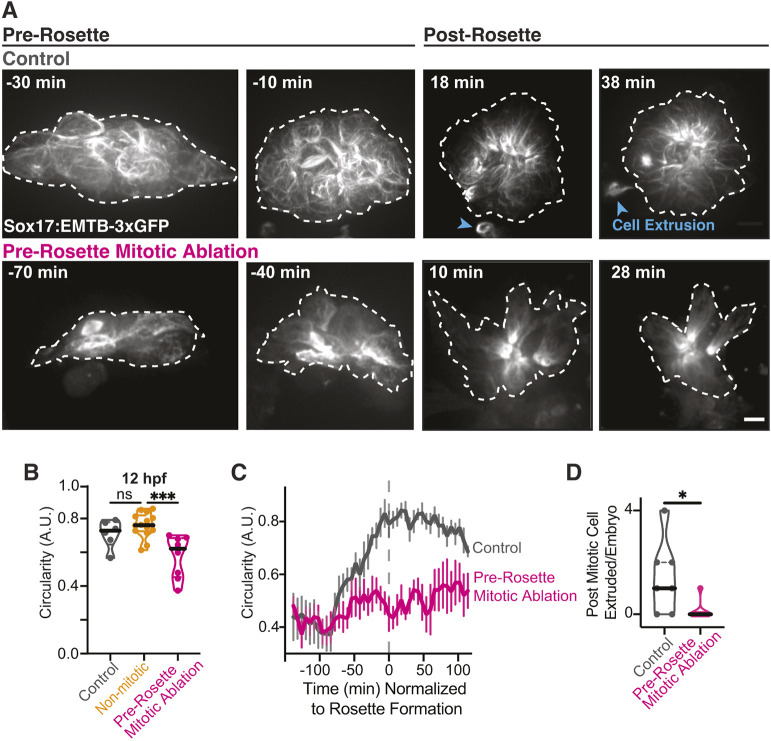
**Pre-rosette mitotic events play an indispensable role in cell packing and subsequent cell extrusion during lumen formation.** (A) Stills from a time-lapse movie of microtubule organization during KV formation (Sox17:EMTB-3xGFP; gray). KVs are outlined (white dashed line) for control (no ablation, top) and pre-rosette mitotic cell ablation conditions (bottom). Time is normalized to rosette formation (0 min); pre-rosette 0 to −70 min, post rosette is 0 to 30 min. Extruded cells are indicated with blue arrowheads and KV outline is highlighted by a white dashed line. Scale bar: 10 μm. See also Movie 4. (B) Truncated violin plot of KV circularity at 12 hpf. Control, non-mitotic cell ablation, and pre-rosette mitotic ablation conditions are shown. Median denoted with black line and quartile denoted by dashed line. *n*≥6 embryos were used across each condition. ***P<0.001 (one-way ANOVA with Dunnett's multiple comparison to non-mitotic cell ablation); ns, not significant. (C) Circularity of KV was measured over time normalized to rosette formation (marked by gray dashed line). Control (no ablation, gray) and ablation of pre-rosette mitotic events (four cells ablated, magenta) are shown. *n*≥6 embryos per condition. Error bars represent s.e.m. (D) Truncated violin plots of post mitotic cell extrusion events per embryo for control (no ablation) and ablation of pre-rosette mitotic events. Median denoted with black line and quartile denoted by dashed line. *n*≥6 embryos per condition. *P<0.05 (unpaired, two-tailed Student's *t*-test).

We found that mitotic cells ended up having an increased rate of extrusion when the KV started to round (Fig. 5E). To test whether early mitotic events are specifically required for KV rounding and whether KV rounding reinforces mitotic extrusion, we used laser ablation in *Sox17:EMTB-3xGFP; h2afx:h2afv-mCherry* embryos (EMTB-3xGFP shown in Fig. 6A) or *Sox17:GFP-CAAX; h2afx:h2afv-mCherry* embryos. Our hypothesis was that early mitotic events are required for KV rounding. To test this, we measured KV circularity under three conditions: control (no mitotic cells ablated), ablation of non-mitotic cells (three to eight cells), and ablation of pre-rosette stage mitotic cells (four cells). Circularity was measured by tracing the KV outline during its development (Fig. 6A, dashed line) and at 12 hpf, when the KV is expected to be more rounded (Fig. 6B). Notably at 12 hpf, under control conditions and in cases where non-dividing cells were ablated before the KV transitioned to the rosette stage (Fig. 6B), the KV achieved comparable levels of rounding. When pre-rosette stage mitotic cells were ablated (Fig. 6B), KV rounding was significantly reduced compared to both non-ablation controls and ablations targeting non-mitotic cells (Fig. 6B). A direct comparison of non-ablation controls to pre-rosette mitotic ablation conditions, normalized to the time of rosette formation, revealed that control KVs exhibited a steady increase in circularity from 100 to 0 min before rosette formation (Fig. 6C). In contrast, this significant increase in circularity was absent under ablation conditions (representative images in Fig. 6A; quantitative analysis in Fig. 6C). These findings suggest that early mitotic events are crucial for the organization of KV cells to transition from an elongated to a rounded structure, whereas non-mitotic cells appear to be dispensable for this transition.

We next investigated whether mitotic extrusion events still occurred under conditions in which KV rounding was disrupted. This was achieved by ablating mitotic events at the pre-rosette stage and comparing to control conditions without ablation (Fig. 6A, blue arrowheads indicate extrusion events in controls; Movie 4). Under ablation conditions, the targeted cells were eventually extruded from the KV and no longer exhibited the h2afx:h2afv-mCherry signal; these extrusions were excluded from our analysis of spontaneous cell extrusion events. When quantifying the number of post-mitotic cell extrusion events per embryo after the rosette stage, we found that under control conditions, each embryo exhibited zero to four extrusion events. In contrast, when pre-rosette stage mitotic events were ablated, extrusion events were rarely observed (Fig. 6D). These findings suggest that extrusion may act as a mechanism to alleviate increased cell packing resulting from KV rounding and lumen formation. When early mitotic events are disrupted, the KV is unable to round; however, subsequent cell divisions can still integrate into the KV and contribute to its development, potentially compensating for the absence of earlier mitotic events. This raises an important question: are early mitotic events essential for proper KV development, or can later events adequately compensate for their loss?

### Early KV mitotic events hold greater significance to KV development compared to later KV mitotic events

**Fig. 7.**
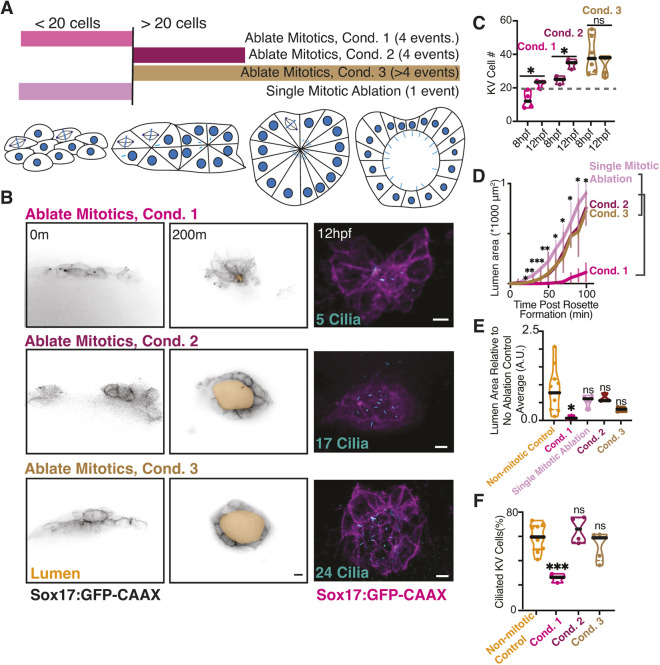
**Early KV developmental mitotic events hold greater significance to KV development compared to later KV mitotic events.** (A) Model illustrating laser ablation conditions during KV development. Pale blue lines represent cilia. (B) Confocal projections taken from a movie acquisition (0-200 min, left; Sox17:GFP-CAAX, inverted gray; lumen, gold; see also Movie 5). The embryos were then fixed at 6SS (12 hpf) and stained for cilia (acetylated tubulin, cyan, right; Sox17:GFP-CAAX in magenta). The number of cilia in each representative image is noted. m, min. Scale bars: 10 μm. (C) Truncated violin plot depicting the number of KV cells at 8 hpf and 12 hpf for ablation conditions 1, 2 or 3 across *n*≥3 embryos (each point represents a single embryo). Median denoted with black line and 20 KV cell threshold demonstrated with gray dashed line. *P<0.05 (unpaired, two-tailed Student's *t*-test); ns, not significant. (D) Lumen area was measured over time for over 100 min for ablation conditions 1, 2, 3, or a single mitotic cell ablation. *n*≥3 embryos measured per condition. Error bars represent s.e.m. *P<0.05, **P<0.01, ***P<0.001 (one-way ANOVA with Dunnett's multiple comparison); ns, not significant. (E,F) Truncated violin plots depicting KV lumen area (12 hpf; E) and percentage of ciliated KV cells (F). *n*≥3 embryos for each condition. *P<0.05, ***P<0.001 (one-way ANOVA with Dunnett's multiple comparison to non-mitotic ablation controls); ns, not significant. A.U., arbitrary units; Cond., condition.

To determine the role and relevance of individual KV mitotic events, we again employed laser ablation to selectively remove mitotic events (Fig. 7A). Temporal characterization of mitotic events involved three ablation conditions, along with two control scenarios: no ablation and ablation of non-mitotic cells (three to eight cells). The mitotic ablation conditions consisted of specific temporal ablation patterns (Fig. 7A): (1) ablating the first four mitotic events when fewer than 20 KV precursor cells were present (Condition 1); (2) ablating four mitotic events when more than 20 KV precursor cells were present (Condition 2); and (3) ablating all mitotic events when more than 20 KV precursor cells were present (Condition 3).

Our studies indicate that the first four KV divisions are crucial for KV development, while later divisions are not essential (Fig. 7B-F). KVs showed an increase in cell number when the first four mitotic events were ablated in embryos with fewer than 20 KV precursor cells (Condition 1; Fig. 7C), as well as when four mitotic events were removed in embryos with more than 20 precursor cells (Condition 2; Fig. 7C). When all mitotic events were ablated in embryos with more than 20 KV precursor cells (Condition 3; Fig. 7C), there was no significant increase in cell number between 8 hpf and 12 hpf. These findings demonstrate that mitotic events are essential for KV cell number expansion during development but also suggest that ablating a subset of mitotic events does not prevent later mitotic events from occurring (Conditions 1 and 2). This observation indicates that KV progenitor cells entering division when there are fewer than 20 total cells are unlikely to undergo multiple rounds of division. This conclusion aligns with our H2B-Dendra-based characterization (Fig. 3F-H), which showed that the progeny of a division event did not divide again.

We quantified lumen formation kinetics under each ablation condition and found that significant defects in both lumen formation kinetics (Fig. 7B,D, Movie 5) and final lumen area (Fig. 7E) occurred only when the first four mitotic events were ablated (Condition 1). In contrast, ablating later mitotic events (Conditions 2 and 3) did not produce these defects. Additionally, lumen formation remained unaffected in control conditions where four to six non-mitotic cells were ablated throughout KV development (Fig. 7E). To determine whether disrupting a single early mitotic event affected lumen formation (Fig. 7A), we ablated one mitotic cell in KV with fewer than 20 cells. No significant defects were observed under this condition (Fig. 7D,E). These findings suggest that while early mitotic events are crucial for lumen development, the loss of a single event can be compensated for by others at this stage. Embryos subjected to ablation and monitoring for lumen formation kinetics (Fig. 7B,D) were fixed at 12 hpf and immunostained for cilia. Like lumen formation defects, significant reductions in the percentage of ciliated cells were observed only when ablating the first four events (Fig. 7B,F). These findings underscore the pivotal role of early KV mitotic events in orchestrating both KV development and overall KV ciliogenesis.

## DISCUSSION

**Fig. 8.**
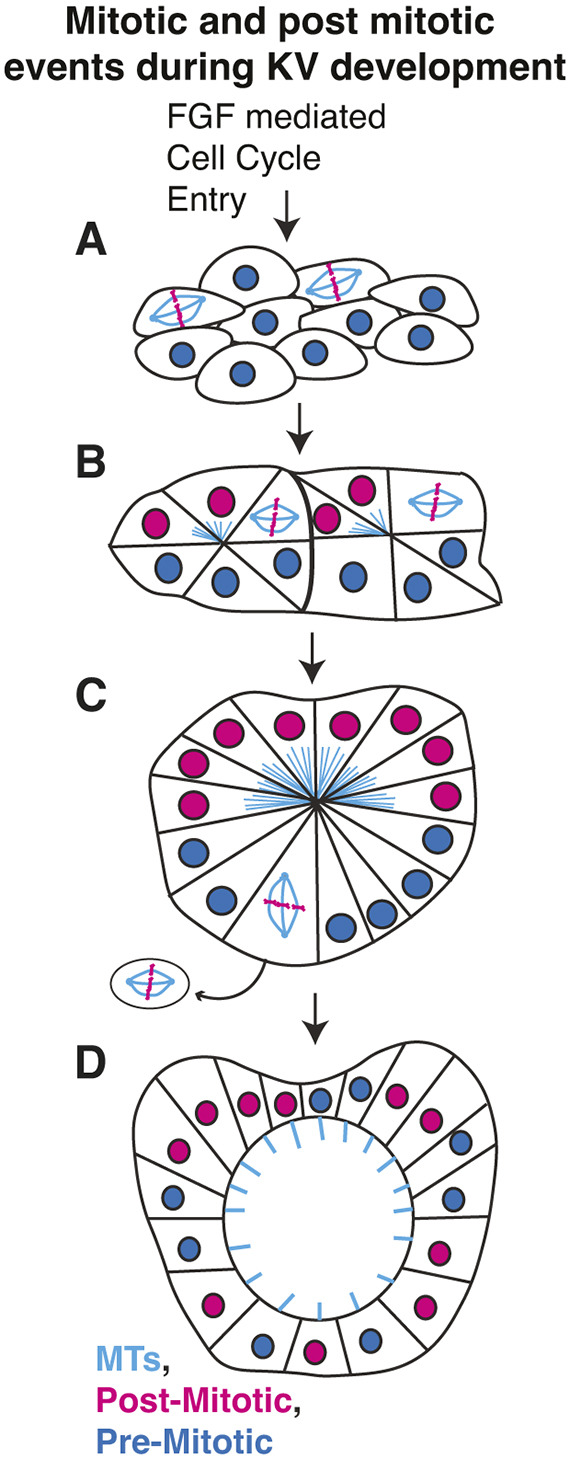
**Model for mitotic and cellular events that occur during KV development.** (A) Anterior KV precursor cells are stimulated by FGF to enter mitosis. (B) KV cells begin to form small rosettes, comprising both post-mitotic cells and those still dividing, with mitotic activity concentrated anteriorly. (C) Cells transition into a single rosette, with anterior post-mitotic cells contributing cytokinetic bridges toward the rosette center. (D) The rosette matures into a ciliated KV with a fluid-filled lumen, and post-mitotic cells become evenly distributed. MTs, microtubules.

Studies of LRO formation have emphasized the importance of LRO progenitors in establishing a rosette-like structure with epithelial-like characteristics prior to lumen formation (Aljiboury et al., 2023; Essner et al., 2005; Gredler and Zallen, 2023). However, the cellular mechanisms leading up to lumen formation within this group of cells remain poorly understood. Here, we present a model describing mitotic and post-mitotic cellular behaviors during KV development (Fig. 8). Disruptions to mitotic events, whether through *pcnt* loss or direct ablation of mitotic cells, result in KV developmental defects. These defects may contribute to increased susceptibility to left-right axis determination defects, such as abnormalities in heart looping (Fig. 1C,D). However, in the case of *pcnt* loss, an alternative possibility is that *pcnt* directly modulates heart development, as suggested by mouse studies (Chen et al., 2014). If this were the case, we would expect more than 50% of embryos to exhibit heart defects, exceeding the rate expected from randomization alone. Since we find that less than 50% of our surviving *pcnt^−/−^* animals present with heart-looping defects, we propose that the defects may arise from deficiencies in KV development. The most direct way to test this hypothesis would be to rescue *pcnt* loss specifically in the KV, an approach we aim to pursue in future studies.

Using novel transgenic lines and cell biological tools, we propose a model in which FGF signaling drives the proliferation of a subset of anterior KV precursor cells (Fig. 8A). Disrupting these divisions, either genetically in *pcnt*-null embryos or via laser ablation, perturbs KV development. Our findings suggest that the progeny of these early divisions integrate into small rosette structures (Fig. 8B), which later coalesce into a larger rosette (Fig. 8C), coinciding with KV rounding prior to lumen formation. Notably, we observed that daughter cells remain connected by cytokinetic bridges, as revealed using the *Sox17:EMTB-3xGFP* transgenic line (Fig. 5A), supporting previous midbody labeling studies (Rathbun et al., 2020a). Within these bridges, we identified bundled microtubules oriented toward the rosette center, structures not detected in earlier studies. Importantly, cytokinetic bridge integrity is essential for lumen formation, as midbody ablation prevents its initiation (Rathbun et al., 2020a). These cytokinetic bridges may serve as focal points for Rab11 vesicle accumulation. Rab11, a small GTPase, facilitates KV lumen formation by directing vesicles containing cargo such as CFTR, a protein that promotes lumen expansion when localized to the apical membrane (Krishnan et al., 2022; Navis et al., 2013; Rathbun et al., 2020a). This raises a crucial question: what roles do these bridges and the microtubule structures at the rosette center play in lumen formation, and why are they essential at this site? Our findings support a testable model in which these structures function as symmetry-breaking events, directing the polarized delivery of key components to coordinate bridge cleavage and lumen formation.

We further found that cells undergoing division as the KV transitions from an elongated configuration (Fig. 8A,B) to a more rounded shape (Fig. 8C) are at higher risk for extrusion from the KV (Fig. 8C). This extrusion likely results from increased cell packing likely driven by the geometric and structural changes occurring during rosette formation. Interestingly, alleviating this packing strain by removing early mitotic events prevents extrusion of later division events. However, when the first four mitotic events are eliminated, when the KV comprises fewer than 20 cells, the KV fails to organize into a traditional rosette-like structure with the KV adopting a round shape. This suggests that these initial divisions play a foundational role in establishing the rosette and enabling subsequent KV morphogenesis. In our model, the KV transitions from a rounded, rosette-like structure where post-mitotic cells are enriched at the anterior of the rosette to a cyst-like structure composed of ciliated cells surrounding a fluid-filled lumen, with post-mitotic cells becoming redistributed throughout the KV (Fig. 8D).

Our findings herein provide important insights into the cellular dynamics of early KV development and point to future studies aimed at uncovering how these early mitotic events contribute to the initiation of the rosette structure, a crucial precursor to lumen formation.

## MATERIALS AND METHODS

### Fish lines

Zebrafish lines were maintained in accordance with protocols approved by the Institutional Animal Care Committee of Syracuse University (IACUC Protocol #18-006). Embryos were raised at 28.5°C and staged (as described by Kimmel et al., 1995). Wild-type and/or transgenic zebrafish lines used for live imaging and immunohistochemistry are listed in Table S1.

### Chemical inhibitors

Dechorionated live embryos were treated with 10 μM XAV939 (Sigma-Aldrich), 0.5 to 5 μM infigratinib (MedChem Express) and 50 μM cyclopamine (Sigma-Aldrich) were prepared. *Tg(Sox17:GFP-CAAX)* transgenic zebrafish embryos were dechorionated on 2% agarose pad dishes and at 50% epiboly incubated with vehicle control (0.4% DMSO, 0.5% methanol) and in listed concentrations of inhibitor diluted in zebrafish embryo water (0.03% sea salt, 1×10^−5^% Methylene Blue, reverse osmosis water) and also taking into account the volume of agarose pad. Embryos were incubated to 8 hpf and 12 hpf and fixed in 4% paraformaldehyde (PFA) (see ‘Immunofluorescence’ section).

### BrdU incorporation

Tg(Sox17:GFP-CAAX) embryos were dechorionated at 50% epiboly, followed by incubation in E3 buffer (5 mM NaCl, 0.17 mM KCl, 0.33 mM CaCl_2_, 0.33 mM MgSO_4_) for 15 min at room temperature. Embryos were then incubated in 10 mM BrdU in E3 buffer containing 12% DMSO and maintained at temperatures between 28.5°C and 30°C until 10 hpf. Following serial dilution washes in E3 medium, the embryos were incubated until 12 hpf and fixed in 4% PFA followed by overnight dehydration in cold methanol at −20°C. Embryos then underwent a sequential rehydration process using methanol to PBST (0.1% Tween 20 in PBS) mixture ratios of 3:1, 1:1 and 1:3 for 5 min each step. Embryos were refixed for 15 min in 4% PFA with 0.5% Triton X-100, followed by acid treatment with 2 N HCl for 1 h and subsequent neutralization in 0.1 M borate buffer (0.1 M boric acid, 0.076 M NaOH) for 20 min. Standard immunofluorescence approaches were then used to label for BrdU (mouse anti-BrdU; 1:100, Sigma-Aldrich,11170376001: BMC9318). See ‘Immunofluorescence’ section and Table S1.

### Plasmids and mRNA for injection experiments

Plasmids were generated using Gibson cloning methods (NEBuilder HiFi DNA assembly Cloning Kit) and maxi-prepped before injection and/or transfection. mRNA was made using the mMESSAGE mMACHINE™ SP6 transcription kit. See Table S1 for a list of plasmid constructs and mRNA used. Injections of one-cell-staged embryos were performed as described by Aljiboury et al. (2021).

### Immunofluorescence

For immunostaining for PH3, acetylated tubulin and GFP, *Tg(Sox17:GFP-CAAX)* transgenic zebrafish embryos were fixed at 8 and 12 hpf using 4% PFA with 0.5% Triton X-100 overnight at 4°C. Embryos were dechorionated after washing with PBST three times. Embryos were blocked in wash solution (1% DMSO, 1% bovine serum albumin, 0.1% Triton X-100) for 1 h at room temperature with gentile agitation. Primary antibody incubation (diluted in wash solution) was carried out overnight at 4°C. Primary antibodies used were: anti-phosphorylated-histone H3 (rabbit) antibody (1:200; Cell Signaling Technology, 9701S), anti-GFP (chicken) (1:300; GeneTex, GTX13970; AB_371416), anti-acetylated tubulin (mouse) (1:300; Sigma-Aldrich, T6793; RRID: AB_477585), anti-GFP (rabbit) (1:300; Molecular Probes, A-11122; AB_221569); see Table S1. Embryos were then washed and incubated with secondary antibodies for 2-4 h at room temperature or overnight at 4°C. Secondary antibodies used were: Alexa Fluor anti-mouse 568 (1:300; Life Technologies, A10037; RRID: AB_2534013), Alexa Fluor anti-chicken 488 (1:300; Fisher Scientific, A11039), Alexa Fluor anti-mouse 647 (1:300; Life Technologies, A31571; RRID: AB_162542), Alexa Fluor anti-rabbit 488 (1:300; Life Technologies, A21206; RRID: AB_2535792). Embryos were stained with DAPI (1 μg/ml) to label nuclei after three washes with wash solution. Embryos were mounted in 2% agarose after washing with PBS. See Table S1.

### Imaging

Fixed or live dechorionated embryos are embedded in low-melting 1.5% agarose (Table S1) with the KV positioned at the bottom of a #1.5 glass-bottom MatTek plate (Table S1) and imaged using a spinning disk confocal microscope or laser scanning confocal microscope. Zebrafish embryos were imaged using a Leica DMi8 inverted microscope equipped with a X-light V2 confocal unit spinning disk equipped with a Visitron VisiFRAP-DC photokinetics system attached to 405 and 355 nm lasers, a Leica SP8 laser scanner confocal microscope (LSCM) and/or a Zeiss LSCM 980 (Carl Zeiss) with an Airyscan 2 detector. The Leica DMi8 is equipped with a Lumencore SPECTRA X, Photometrics Prime-95B sCMOS camera and 89 North-LDi laser launch. VisiView software was used to acquire images. Optics used with this unit were: HC PL APO ×40/1.10W CORR CS2 0.65 water immersion objective, HC PL APO ×40/0.95 NA CORR dry objective and HCX PL APO ×63/1.40-0.06 NA oil objective. The SP8 laser scanning confocal microscope is equipped with an HC PL APO ×20/0.75 IMM CORR CS2 objective, HC PL APO ×40/1.10 W CORR CS2 0.65 water objective and HC PL APO ×63/1.3 Glyc CORR CS2 glycerol objective. LAS-X software was used to acquire images. The Zeiss LSM 980 is equipped with a T-PMT, GaASP detector, MA-PMT, and Airyscan 2 multiplex with 4Y and 8Y. Optics used with this unit are PL APO ×63/1.4 NA oil DIC. Zeiss Zen 3.2 was used to acquire the images. A Leica M165 FC stereomicroscope equipped with DFC 9000 GT sCMOS camera was used for staging and phenotypic analysis of zebrafish embryos.

### Photoconversion

*Tg(Sox17:GFP)* embryos were injected with 75 ng of Dendra-H2B mRNA (see methods in Aljiboury et al., 2021). The embryos were incubated at 30°C until 8 hpf, dechorionated and embedded in 1.5% agarose. To photoconvert H2B-Dendra, 405 nm laser beam was applied to a ROI placed over a mitotic plane. The 405 nm laser was used at 8 mW power with two FRAP cycles at 50 ms/pixel dwell time. Time-lapse movies were captured using 470 nm and 555 nm lasers to capture green and red emission from non-photoconverted and photoconverted, respectively. *z*-stacks were acquired with a 2 μm step size every 2 min until 12 hpf.

### Laser ablation

*Tg(Sox17:GFP-CAAX; h2afx:h2afv-mCherry)*, *Tg(Sox17:EMTB-3XGFP)* or *Tg(Sox17:EMTB-3xGFP; h2afx:h2afv-mCherry)* dechorionated live embryos were imaged on a spinning disk microscope with VisiView kinetics unit starting at 6 hpf.

#### Mitotic ablation

Time-lapse movies capturing GFP-CAAX or EMTB-3xGFP and h2afx:h2afv-mCherry used 470 nm and 555 nm lasers, respectively, across a *z*-stack with a 2 μm step size every 2 min until 12 hpf. KV mitotic events were ablated using a 355 nm pulsed laser operating at 65% power. The laser was applied within a ROI over mitotic cell for two cycles of 50 ms/pixel. Non-mitotic ablation control involved ablating a region containing three to eight interphase cells of the KV.

### Image and data analysis

Images were processed using Fiji/ImageJ and Imaris (Bitplane). Graphs and statistical analysis were produced using Prism 9 software. Movies were created using Fiji/ImageJ or IMARIS. All time-lapse movie projections are registered using Fiji. For the percentage of ciliated KV cells, the number of cells with cilia was counted and represented as a percentage over the total number of cells in the cyst forming tissue.

#### Mitotic index and anterior versus posterior mitotic event calculation

For mitotic index, the number of mitotic cells (PH3 positive) in the KV was divided by the total number of KV cells (DAPI and Sox17:GFP-CAAX positive), resulting in a percentage of mitotic cells out of the entire population. To calculate the fraction of mitotic events in the anterior versus posterior regions, anterior and posterior KV mitotic cell number was divided by the total number of mitotic cells in the KV.

#### KV AR

KV AR was calculated from a movie projection of Sox17:EMTB-3xGFP embryos from 7 to 12 hpf. Images were acquired at a maximum of 2- to 3-min intervals. For each captured frame, the AR was calculated using Fiji/ImageJ. The longest axis over the shortest axis was determined giving the AR over time.

#### KV volume

Imaris surface renderings were generated using a manual surface protocol, in which fluorescence ROIs were outlined using the isoline function for each *z*-plane at 10-min intervals. Surface renderings of the entire KV and its lumen were created separately. Once surface rendering was completed for each KV, the whole volume and lumen volume were pseudocolored, and each frame was captured. The volume of each surface rendering was measured and recorded using the built-in statistics function of Imaris. The KV volume was then determined by subtracting the lumen volume from the total KV volume. KV volume was normalized by dividing KV volume at each time to the time point with the highest volume of the same KV.

#### Angular velocity of the spindle

The metaphase-derived spindle angle relative to the KV's longest axis was measured over time. To do this, a line was positioned along the longest KV axis in relation to a second line that passed through the two spindle poles and the longest axis of a KV mitotic spindle. The ImageJ/Fiji angle tool calculated the angle at which the two lines intersect providing a spindle angle (∠Spindle). This spindle angle was calculated from metaphase to anaphase completion. The angular velocity was measured by taking the difference of the identified spindle angle across consecutive time point 1 (∠Spindle_T1_) and time point 2 (∠Spindle_T2_) and dividing the difference by time interval (T_1_-T_2_):
(1)

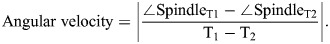
Data were presented by averaging spindle angular velocity from each mitotic event across 20 min bins for the duration of KV development from 7 to 12 hpf.

#### Circularity

KV circularity was calculated from a movie projection of Tg(Sox17:EMTB-3xGFP) or Tg(Sox17:GFP-CAAX; h2afx:h2afv-mCherry) embryos from 7 to 12 hpf. The KV outline was manually tracked every 6 min to determine the perimeter and area. The KV circularity was calculated by 4π times the area divided by the squared perimeter:⁠
(2)

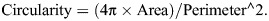


#### Lumen area

Volumetric projected images of the KV, oriented to display the largest perimeter in view, were used to trace the lumen and calculate its area using either Imaris or Fiji. Where applicable, values were normalized to the control mean by dividing each lumen area by the mean value of the control lumens.

### Statistical analysis

Prism 9 software was used for all graph preparations, which include all individual data points across embryos and clutches denoted by the color and size of points, respectively, and as noted in legends. These plots were presented as violin, floating bar, and a modified scatter plot that is termed a ‘super-plot’ (Lord et al., 2020) to denote individual embryos across clutches. Unpaired, two-tailed *t*-tests for pairwise numerical differences in mean and one-way ANOVA were performed when comparing greater than two groups using Prism 9 software. *P*-values are represented as: ****P<0.0001, ***P<0.001, **P<0.01, *P<0.05; ns, not significant. All *n* values and statistical analysis information for each figure are included in Table S2.

## Supplementary Material



10.1242/develop.204687_sup1 Supplementary information

Table S1. Key resources table

Table S2. Detailed statistical analysis of results reported in this study.
