# Supplementary material for: Specific mitotic events drive left-right organizer development
Source: Development. 2025 May 19;152(10):dev204687. doi: 10.1242/dev.204687 (PMC12148018; doi:10.1242/dev.204687)
Supplement: Supplementary information [file develop-152-204687-s1.pdf]

Table S1. Key resources table

Available for download at
https://journals.biologists.com/dev/article-lookup/doi/10.1242/dev.204687#supplementary-data

Table S2. Detailed statistical analysis of results reported in this study.

Available for download at
https://journals.biologists.com/dev/article-lookup/doi/10.1242/dev.204687#supplementary-data

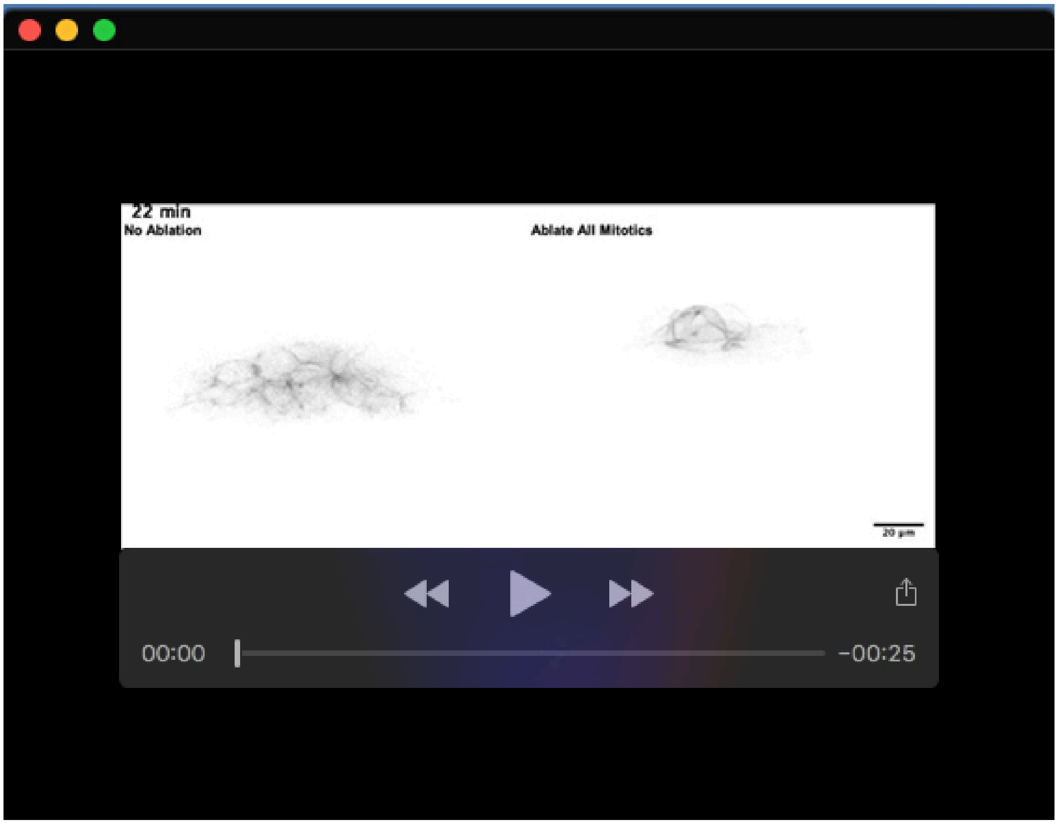

Movie 1. Mitotic events are required for KV formation. Related to Fig. 2C.
Live confocal video showing lumen formation from a Sox17:GFP-CAAX, H2afx:h2afv-mCherry embryos when mitotic events are ablated compared to no ablation control. Sox17:GFP-CAAX (inverted gray) shown. Scale bar, 10 µm.

Development • Supplementary information

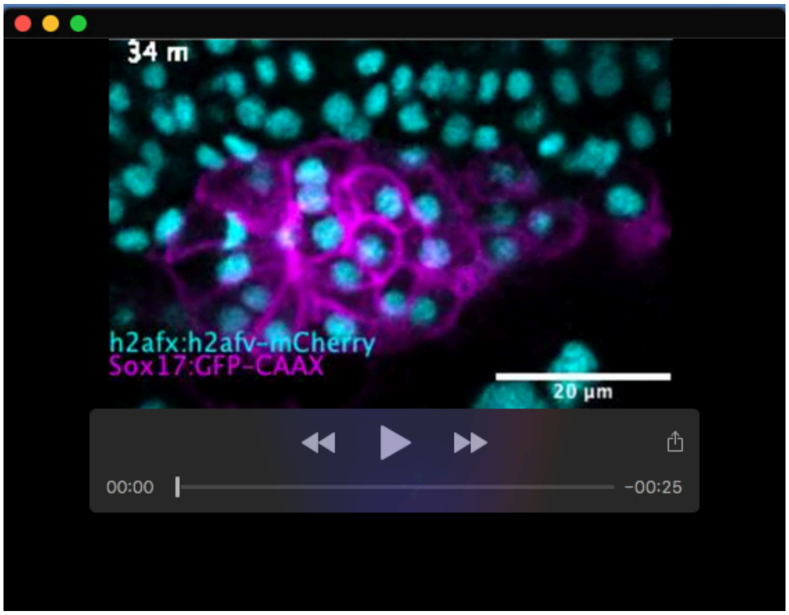

Movie 2. Identification of a pre-lumen enriched KV mitotic events. Related to Fig. 3A. Live confocal registered video showing mitotic events within KV in a Sox17:GFP-CAAX; H2afx:h2afv-mCherry embryo. KV cell plasma membranes marked by magenta (Sox17:GFP-CAAX) and nuclei (cyan, H2afx:h2afv-mCherry). Scale bar, 20 µm.

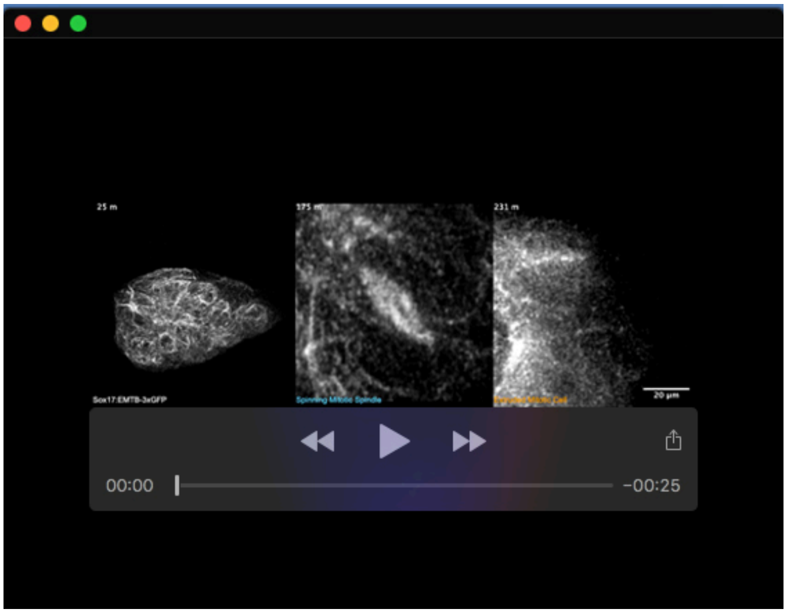

Movie 3. KV spindles stably align until the KV starts rounding, then spindles spin and are extruded. Related to Fig. 5A-B.

Live confocal video showing microtubule (grey, Sox17:EMTB-3xGFP) in KV (left). Highlighted from KV video on left is a spinning spindle (middle) and cell extrusion event (right). Scale bar, 20 µm.

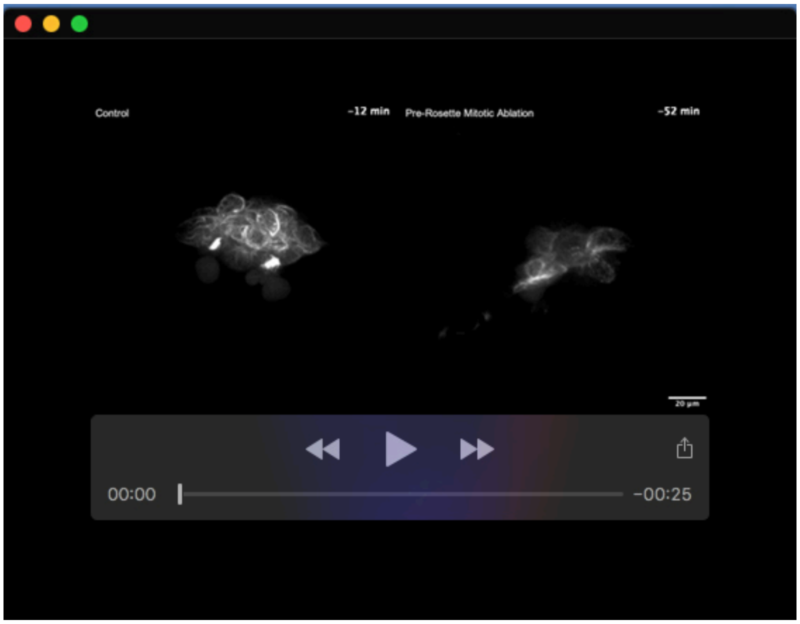

Movie 4. Pre-rosette mitotic events play indispensable role in cell packing during lumen formation. Related to Fig. 6A.

Live confocal video showing microtubule (grey, Sox17:EMTB-3xGFP) in non-ablation controls (left) and pre-rosette mitotic ablated (4 events, right). Scale bar, 20 µm.

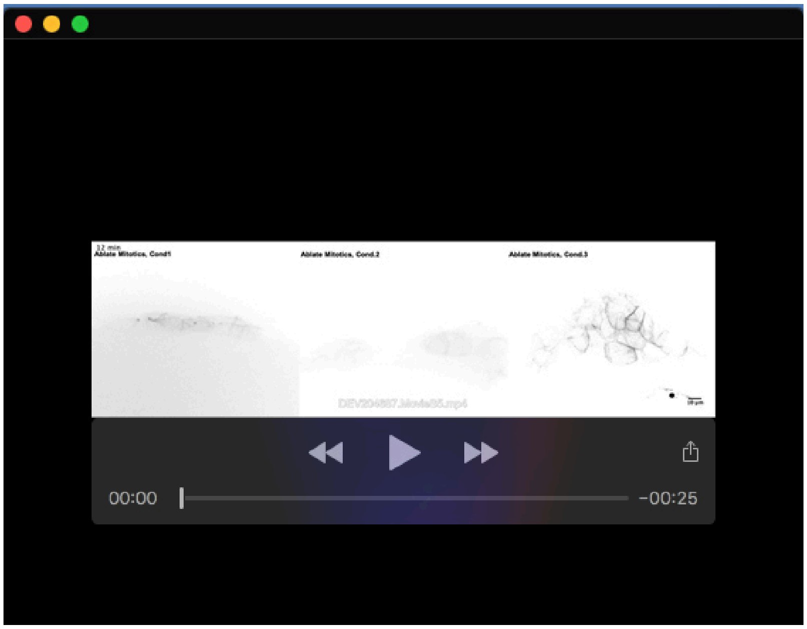

Movie 5. Early KV developmental mitotic events hold greater significance to KV development. Related to Fig. 7B.

Live confocal video showing lumen formation from a Sox17:GFP-CAAX; H2afx:h2afv-mCherry embryos when mitotic events are ablated at various conditions (Cond.1, 2, and 3, refer to **Fig. 7A**). Sox17:GFP-CAAX (inverted gray) shown. Scale bar, 10 µm.
