# Supplementary material for: Specific mitotic events drive left-right organizer development
Source: Development. 2025 May 19;152(10):dev204687. doi: 10.1242/dev.204687 (PMC12148018; doi:10.1242/dev.204687)
Supplement: Supplementary file 1 [file develop-152-204687_review_history.pdf]

Specific mitotic events drive left-right organizer development

Yan Wu, Yiling Lan, Favour Ononiwu, Abigail Poole, Kirsten Rasmussen, Jonah Da Silva, Abdalla Wael Shamil, Li-En Jao and Heidi Hehnly

DOI: 10.1242/dev.204687

Editor: Liz Robertson

Review timeline

Original submission:	30 January 2025
Editorial decision:	24 February 2025
First revision received:	31 March 2025
Accepted:	8 April 2025

Original submission

First decision letter

MS ID#: dev.204687

MS TITLE: Specific Mitotic Events Drive Left-Right Organizer Development

AUTHORS: Yan Wu, Yiling Lan, Favour Ononiwu, Abigail Poole, Kirsten Rasmussen, Jonah Da Silva, Abdalla Wael Shamil, Li-En Jao and Heidi Hehnly

Dear Dr Hehnly,

I have now received all the referees reports on the above manuscript, and have reached a decision. The referees' comments are appended below, or you can access them online: please go to .

The overall evaluation is positive and we would like to publish a revised manuscript in Development, provided that the referees' comments can be satisfactorily addressed. Please attend to all of the reviewers' comments in your revised manuscript and detail them in your point-by-point response. If you do not agree with any of their criticisms or suggestions explain clearly why this is so. If it would be helpful, you are welcome to contact us to discuss your revision in greater detail. Please send us a point-by-point response indicating your plans for addressing the referees' comments, and we will look over this and provide further guidance.

Reviewer 1

Advance summary and potential significance to field

In the study by Wu et al (dev.204687), the authors studied the formation of the Kupffers vesicle (KV) during early embryogenesis in pericentrin -/- zebrafish. They found that mitotic cell divisions at 8hpf, prior to the development of the lumen lined with ciliary cells, were critical for proper formation of this structure. This was was further supported by ablating mitotic cells in control embryos during this period of cell division. If mitotics were ablated the embryos formed a small KV with a small lumen containing decreased numbers of ciliated cells. The authors show that early mitotic divissions are important for developing a proper KV and lumen and that this process is less dependent on later cell divisions. I found this study to be interesting and stimulating for further research. The timing and spatial reorganization of the cell division behavior was descriptive but

© 2025. Published by The Company of Biologists under the terms of the Creative Commons Attribution License (https://creativecommons.org/licenses/by/4.0/).

novel. This lab is well known for using skilled imaging to study early divisions and pattern formation in zebrafish embryos to fill an important niche in the zebrafish early development research world.

Comments for the author

The authors spend a lot of time describing pericentrin in the early part of the manuscript but then very little time studying it overall. Only the Figure 1 uses th pcnt-/- mutant. This makes the manuscript seem unbalanced. I would rewrite more clearly how the pcnt -/- mutant sets up the rest of the experiments. Just something like, "the decrease in mitotic cells led us to study this in more detail. To isolate mitotic events we performed the rest of our experiments in the pcnt+/+ background." Just to be sure to bluntly inform the reader early on that there will be no more pericentrin!

Does a mitotic "cohort" stay physically attached during movement. As through a midbody?

Spindle spinning suggests a contribution by the actin cytoskeleton. Do the authors see, in any of their existing movies, any suggestion of membrane blebbing or excitability during the time at which spindles would be spinning (163 min. in Fig. 5A). While I believe that a foray into the actin cytoskeleton is beyond the scope of this study, you might already have some preliminary data along these lines. This might be discernible in existing movies where membranes are labeled.

Figure 7A is hard to initially interpret, although it is well explained in the text. It superficially appears like Cond 1 is labeling the magenta, rather than the pink bar. However, I do not have a tidy way to fix this. Perhaps the text could be the same color as the bar.

Page 3, line 8-9:
It says: "Compromised formation of LRO, such as disruption in lumen formation [4,5] or its cells having ciliary motility (e.g., mutations in the axonemal dynein motor [6-8])".
Do the authors mean ... or its cells having abnormal ciliary motility".

Page 4, lines 5-7:
The text says: "... 3D cell culture models do not recapitulate the mesenchymal to epithelial transition…" I think it would be useful to say that those MDCK cells are already epithelial cells. Those 3D cultures can not recapitulate MET because those cells are already epithelial, is that right? As far as I know, it is possible to have 3D cultures of mesenchymal cells for MET. There is this paper: https://pubmed.ncbi.nlm.nih.gov/30474817/

where they explore the possibilities of MET for breast cancer. It's not very important, but I wanted to let you know in case you want to change the sentence. However, this is not critical.

Page 5, lines 14-15, Fig 1B:
Not a big deal, but would it be possible to put more tick marks in the X and Y axis of Fig. 1B? That way it would be easy to see that most pcnt KO embryos die within the first 12h? In fact, instead of "most embryos" it would be better to put exactly the number of embryos dying within those 12h since you have these data.

Fig 1C: the myosin light chain gene cmlc2 is currently named myl7. I think that's the official and current name.

Page 5, line 24: How is that just 28% of pcnt KO embryos have heart looping defects? In the line 28 and 29 it also said that we cannot rule out the possibility that pcnt directly modulates heart development. I agree with that statement, but wouldn't you expect a higher percentage of embryos with heart looping defects if there was a direct modulation by pcnt? I think the sentence of lines 28 and 29 would fit better in the discussion.

Page 6, line 3: It should say "phosphor-histone H3".

Page 6, line, 28, and page 7, line 1: It would be better if the mCherry was the pink color and the GFP the cyan. But it's not very important.

© 2025. Published by The Company of Biologists under the terms of the Creative Commons Attribution License (https://creativecommons.org/licenses/by/4.0/).

Page 8, lines 10, Fig. 3B: It would be helpful to have more tick marks in Fig. 3B. It would be very helpful to know where those measured points are.

Page 9, line 22-23: It should say: "Since early mitotic events occurred in the anterior half of the developing KV (Fig 1 and Fig 3),..."

Page 10, lines 1-4: I would change the order of the first two sentences. I would put it like this: "The FGF, Wnt, and Shh signaling pathways were inhibited using infigratinib, XAV939, and cyclopamine, respectively, at concentrations within the ranges previously reported in the literature [35-38]. We found that blocking FGF signaling, but not Wnt or Shh signaling, caused significant defects to lumen formation (Fig 4B-D)." It makes more sense to me to first mention the drugs that you are going to use and then talk about the result.

Page 10, line 6: I would say here that even at 0.5 uM you can see a reduction of even a half of lumen area. That's also a very dramatic effect.

Page 10, line 8, Fig. 4E: What concentration of FGF inhibitors is this? 1 uM? I assume so, but it would be nice to write it here.

Page 10, Line 17-18: I know that this is not the scope of this paper, but do you know how the FGF signaling is affecting primarily the anterior side? I'm not asking for experiments, but it would be an interesting topic for the discussion.

Page 11, line 1: where it says ... stably positioned their spindles around 8 hpf when the KV has a more elongated shape..." it would be good to have an idea of the orientation of the spindles. The figure 5A clearly shows that one spindle is not oriented in an anterior-posterior direction, is that the case for all the spindles?

Page 11, line 10: In the sentence ... but were unable to assess MT-dynamics at the time." It would be important to change "MT-dynamics" by "spindle-dynamics". This is because in this manuscript there are no classical measurements of MT-dynamics like MT-polymerization rates, catastrophes, rescues, etc. So, change it for "spindle-dynamics" seems more accurate.

Page 11, last paragraph and page 12, first paragraph: This is a very interesting piece of data. It seems that between -60 and 0 min there is an increase in the spindle angular velocity. This time also correspond with the increase in mitotic cells seeing in Fig. 3B. You also see an increase in KV volume, AR and circularity. Why would an increase in the number of mitotic cells would also increase the spindle angular velocity? Any idea? Is it possible that the stabilization of the mitotic spindle is linked to external forces within the KV? Is the morphology of those cells different? This would be an interesting topic for discussion.

Page 13, line 11: Where it says ... minutes before rosette formation", I would add (Fig. 6C) after the word formation.

Page 13, lines 26-27: "These findings suggest that extrusion may act as a mechanism to alleviate increased cell packing resulting from KV cell rounding." Wouldn't it be more accurate to say ... resulting from KV rounding and lumen formation"? Just before lumen opening there is an increase in KV volume, circularity and number of mitotic cells, so it makes sense the extrusion as a mechanism to alleviate cell packing, but just if a lumen is created. Without a lumen, you could have a KV rounding without increase cell packing because you would have more space. I think it is the creation of the lumen and rounding together that triggers this effect of increase cell packing.

Figure 3B: The X axis in the graph says Time (hours), however the figure legend says "120 min video".

References:
Page 35, line 31: The reference 23 seems to have a duplicated title.

© 2025. Published by The Company of Biologists under the terms of the Creative Commons Attribution License (https://creativecommons.org/licenses/by/4.0/).

Reviewer 2

Advance summary and potential significance to field

In this manuscript, Wu et al. identify novel roles for mitotic events in the formation of the zebrafish left-right organizer, known as Kupffer's Vesicle. Building off their prior work, in which they showed that cytokinesis promotes apical membrane specification in KV, here they study early mitotic events and their roles in KV morphogenesis. Through genetic and chemical perturbations and laser ablations, the authors find that fgf signaling promotes mitosis in a small population of anteriorly positioned KV cells at early stages. Surprisingly, these early mitotic events of just a few cells play an outsized role in morphogenesis and lumen formation relative to those of later stages. Finally, the authors find that early dividing KV cells have a unique function in cell rearrangements that are necessary for growth and morphogenesis of the organ. Overall, the ablation experiments are well designed, the main conclusions are supported by the data presented, and the findings will be of interest to developmental biologists.

Comments for the author

Essential revisions:

1) Ablation of early mitotics
a. Have the authors performed experiments in which only a small proportion of early mitotics are ablated rather than all of them (for example, ablating only 1 or 2 of the first 4 mitotics), and does this lead to an intermediate lumen phenotype? This control would help to more convincingly show the morphogenetic phenotypes are not an artifact of KV ablation at the early stage. Alternatively, this concern could be addressed if the authors have performed a control experiment of ablating non-mitotic cells at the same exact stage of the early mitotic ablations (during the first 4 divisions).

2) There appears to be an error of the x axis of Fig. 3B.

Additional minor comments:

3) Pericentrin mutants
a. Given that a significant proportion of pcnt mutants die during the experimental window (by 12 hpf), can the authors clarify whether the analyses in Fig.1 E-H performed on mutants that did not show widespread necrosis?
b. To show that loss of pericentrin function in KV is responsible for the phenotypes shown in Fig.1 C-H, a rescue experiment would be needed, for example by expressing sox17:pcnt in mutants. I don't consider this essential because the ablation experiments generally support the conclusion, but this should be noted.

4) Relationship of early and late mitotic events to trafficking of CFTR
a. It would be helpful to briefly discuss their results in the context of their prior work on mitosis in KV. Although this is alluded to in the discussion section, can the authors comment on how early versus late mitotic events might relate to the apical positioning of Rab11 and CFTR that is needed for lumen expansion?

5) Fgf
a. It would be helpful for the authors to cite a paper showing fgf signaling activity in DFCs and in KV (Molina et al., 2007. BMC Dev Biol).

First revision

Author response to reviewers' comments

© 2025. Published by The Company of Biologists under the terms of the Creative Commons Attribution License (https://creativecommons.org/licenses/by/4.0/).

Reviewer 1: SUMMARY OF THE ADVANCE MADE IN THIS PAPER AND ITS POTENTIAL SIGNIFICANCE TO THE FIELD

In the study by Wu et al (dev.204687), the authors studied the formation of the Kupffers vesicle (KV) during early embryogenesis in pericentrin -/- zebrafish. They found that mitotic cell divisions at 8hpf, prior to the development of the lumen lined with ciliary cells, were critical for proper formation of this structure. This was was further supported by ablating mitotic cells in control embryos during this period of cell division. If mitotics were ablated the embryos formed a small KV with a small lumen containing decreased numbers of ciliated cells. The authors show that early mitotic divissions are important for developing a proper KV and lumen and that this process is less dependent on later cell divisions. I found this study to be interesting and stimulating for further research. The timing and spatial reorganization of the cell division behavior was descriptive but novel. This lab is well known for using skilled imaging to study early divisions and pattern formation in zebrafish embryos to fill an important niche in the zebrafish early development research world.

We thank the reviewer for their positive review of the paper and their helpful suggestions. We have incorporated or addressed all the suggestions below.

SUGGESTIONS TO AUTHORS
The authors spend a lot of time describing pericentrin in the early part of the manuscript but then very little time studying it overall. Only the Figure 1 uses th pcnt-/- mutant. This makes the manuscript seem unbalanced. I would rewrite more clearly how the pcnt -/- mutant sets up the rest of the experiments. Just something like, "the decrease in mitotic cells led us to study this in more detail. To isolate mitotic events we performed the rest of our experiments in the pcnt+/+ background." Just to be sure to bluntly inform the reader early on that there will be no more pericentrin!
We have incorporated your suggestion and have highlighted it in the text.

Does a mitotic "cohort" stay physically attached during movement. As through a midbody?
Our data suggest that postmitotic cells may maintain connectivity through cytokinetic bridges because they remain clustered together until lumen formation (Fig. 3F). Our previous work demonstrated that post-mitotic cells are interconnected by cytokinetic bridges that orient toward the rosette center, and the bridges do not abscise until the KV lumen decides to open (Rathbun et al. 2020).

Spindle spinning suggests a contribution by the actin cytoskeleton. Do the authors see, in any of their existing movies, any suggestion of membrane blebbing or excitability during the time at which spindles would be spinning (163 min. in Fig. 5A). While I believe that a foray into the actin cytoskeleton is beyond the scope of this study, you might already have some preliminary data along these lines. This might be discernible in existing movies where membranes are labeled.
We thank the reviewer for this comment. We have indeed been considering the relevance of actin to the phenomenon we observed. While we have investigated the actin cytoskeleton organization and dynamics in the KV our work is currently too preliminary to understand if there is a correlation between spindle angular velocity and actin organization. We are currently developing new zebrafish lines and refining experimental strategies to adequately address this question.

Figure 7A is hard to initially interpret, although it is well explained in the text. It superficially appears like Cond 1 is labeling the magenta, rather than the pink bar. However, I do not have a tidy way to fix this. Perhaps the text could be the same color as the bar.
We thank the reviewer for this observation. In response, we have revised the model labeling to enhance clarity.

Page 3, line 8-9:
It says: "Compromised formation of LRO, such as disruption in lumen formation [4,5] or its cells having ciliary motility (e.g., mutations in the axonemal dynein motor [6-8])".
Do the authors mean "... or its cells having abnormal ciliary motility".
Thank you for your suggestion. We have revised the sentence accordingly.

Page 4, lines 5-7:

© 2025. Published by The Company of Biologists under the terms of the Creative Commons Attribution License (https://creativecommons.org/licenses/by/4.0/).

The text says: "...3D cell culture models do not recapitulate the mesenchymal to epithelial transition..." I think it would be useful to say that those MDCK cells are already epithelial cells. Those 3D cultures can not recapitulate MET because those cells are already epithelial, is that right? As far as I know, it is possible to have 3D cultures of mesenchymal cells for MET. There is this paper: https://pubmed.ncbi.nlm.nih.gov/30474817/

where they explore the possibilities of MET for breast cancer. It's not very important, but I wanted to let you know in case you want to change the sentence. However, this is not critical.
We thank the reviewer for the helpful suggestion. We have revised the sentence to clarify that, as MDCK cells are inherently epithelial, their 3D culture does not recapitulate the mesenchymal-to-epithelial transition.

Page 5, lines 14-15, Fig 1B:
Not a big deal, but would it be possible to put more tick marks in the X and Y axis of Fig. 1B? That way it would be easy to see that most pcnt KO embryos die within the first 12h? In fact, instead of "most embryos" it would be better to put exactly the number of embryos dying within those 12h since you have these data.
We thank the reviewer for this suggestion. We have updated Figure 1B and 3B by adding additional tick marks to the X and Y axes for enhanced clarity. Furthermore, we replaced the phrase “most embryos” with the precise percentage of embryos that die.

Fig 1C: the myosin light chain gene cmlc2 is currently named myl7. I think that's the official and current name.
We have changed cmlc1 to myl7 in text and Figure 1 and Figure 1 legend.

Page 5, line 24: How is that just 28% of pcnt KO embryos have heart looping defects? In the line 28 and 29 it also said that we cannot rule out the possibility that pcnt directly modulates heart development. I agree with that statement, but wouldn't you expect a higher percentage of embryos with heart looping defects if there was a direct modulation by pcnt? I think the sentence of lines 28 and 29 would fit better in the discussion.
If the KV were completely abolished, we would expect heart looping to be randomized, resulting in approximately 50% normal and 50% abnormal cases. However, since the KV is not entirely absent but retains a significantly reduced lumen, it may still provide sufficient function to support normal heart development in 72% of surviving embryos, though this remains significantly below control levels. We have incorporated aspects of this in the results and explanation into the discussion section of the manuscript.

Page 6, line 3: It should say "phosphor-histone H3".
Fixed. We spelled out phosphorylated for clarity

Page 6, line, 28, and page 7, line 1: It would be better if the mCherry was the pink color and the GFP the cyan. But it's not very important.

Page 8, lines 10, Fig. 3B: It would be helpful to have more tick marks in Fig. 3B. It would be very helpful to know where those measured points are.

The data points are 20 min bins of the percentage of cells that were mitotic from videos with 2 min acquisition data sets (Movie 2 is one n-value) during lumen formation. This has been clarified in the figure legend. Additional tick marks have been added.

Page 9, line 22-23: It should say: "Since early mitotic events occurred in the anterior half of the developing KV (Fig 1 and Fig 3),..."
Fixed

Page 10, lines 1-4: I would change the order of the first two sentences. I would put it like this: "The FGF, Wnt, and Shh signaling pathways were inhibited using infigratinib, XAV939, and cyclopamine, respectively, at concentrations within the ranges previously reported in the literature [35-38]. We found that blocking FGF signaling, but not Wnt or Shh signaling, caused significant defects to lumen formation (Fig 4B-D)." It makes more sense to me to first mention the drugs that you are going to use and then talk about the result.

© 2025. Published by The Company of Biologists under the terms of the Creative Commons Attribution License (https://creativecommons.org/licenses/by/4.0/).

Fixed, refer to highlighted text.

Page 10, line 6: I would say here that even at 0.5 uM you can see a reduction of even a half of lumen area. That's also a very dramatic effect.
Edited text to reflect this.

Page 10, line 8, Fig. 4E: What concentration of FGF inhibitors is this? 1 uM? I assume so, but it would be nice to write it here.
Done.

Page 10, Line 17-18: I know that this is not the scope of this paper, but do you know how the FGF signaling is affecting primarily the anterior side? I'm not asking for experiments, but it would be an interesting topic for the discussion.
We appreciate the reviewer's insightful question. Although elucidating this mechanism is beyond the current study's scope, one hypothesis is that a localized source of FGF—potentially FGF8, as suggested by a preliminary scRNA-seq data—might generate a diffusion gradient that primarily affects cells in the anterior region. This possibility represents an intriguing topic for future investigation and discussion.

Page 11, line 1: where it says "...stably positioned their spindles around 8 hpf when the KV has a more elongated shape..." it would be good to have an idea of the orientation of the spindles. The figure 5A clearly shows that one spindle is not oriented in an anterior-posterior direction, is that the case for all the spindles?
We appreciate the reviewer's comment. In the revised manuscript, we have clarified that "stably positioned spindles" refers to those that exhibit minimal wobbling and significantly reduced spindle velocity compared to spindles observed during the rosette stages. Importantly, this definition is based on dynamic stability rather than a fixed anterior-posterior orientation, as Figure 5A illustrates that not all spindles necessarily align along the anterior-posterior axis. Refer to edited and highlighted text where we have tried to clarify this point.

Page 11, line 10: In the sentence "...but were unable to assess MT-dynamics at the time." It would be important to change "MT-dynamics" by "spindle-dynamics". This is because in this manuscript there are no classical measurements of MT-dynamics like MT-polymerization rates, catastrophes, rescues, etc. So, change it for "spindle-dynamics" seems more accurate.
Thank you for the suggestion, we have edited the text accordingly.

Page 11, last paragraph and page 12, first paragraph: This is a very interesting piece of data. It seems that between -60 and 0 min there is an increase in the spindle angular velocity. This time also correspond with the increase in mitotic cells seeing in Fig. 3B. You also see an increase in KV volume, AR and circularity. Why would an increase in the number of mitotic cells would also increase the spindle angular velocity? Any idea? Is it possible that the stabilization of the mitotic spindle is linked to external forces within the KV? Is the morphology of those cells different? This would be an interesting topic for discussion.
We appreciate the reviewer's insightful question regarding the potential link between an increase in mitotic cells and spindle angular velocity. We agree that spindle rotation may result from external forces exerted upon and potentially within the KV during this stage of development. Our data suggest that increased spindle dynamics are correlated with KVs transitioning to a rounded structure, implying that the combination of increased mitotic events coupled with overall KV shape changes may cause biomechanical constraints that influence spindle positioning dynamics.
We are excited to follow up on these ideas in future studies, where we will investigate actin dynamics and cell-cell contacts during mitotic events to further elucidate the mechanisms governing spindle orientation within the KV.

Page 13, line 11: Where it says "... minutes before rosette formation", I would add (Fig. 6C) after the word formation.
Done.

Page 13, lines 26-27: "These findings suggest that extrusion may act as a mechanism to alleviate increased cell packing resulting from KV cell rounding." Wouldn't it be more accurate to say "... resulting from KV rounding and lumen formation"? Just before lumen opening there is an increase in

© 2025. Published by The Company of Biologists under the terms of the Creative Commons Attribution License (https://creativecommons.org/licenses/by/4.0/).

KV volume, circularity and number of mitotic cells, so it makes sense the extrusion as a mechanism to alleviate cell packing, but just if a lumen is created. Without a lumen, you could have a KV rounding without increase cell packing because you would have more space. I think it is the creation of the lumen and rounding together that triggers this effect of increase cell packing.
Agree. We have edited the text to clarify these points.
Figure 3B: The X axis in the graph says Time (hours), however the figure legend says "120 min video".
Edited the graph accordingly.

References:
Page 35, line 31: The reference 23 seems to have a duplicated title.
Fixed

Reviewer 2: SUMMARY OF THE ADVANCE MADE IN THIS PAPER AND ITS POTENTIAL SIGNIFICANCE TO THE FIELD
In this manuscript, Wu et al. identify novel roles for mitotic events in the formation of the zebrafish left-right organizer, known as Kupffer's Vesicle. Building off their prior work, in which they showed that cytokinesis promotes apical membrane specification in KV, here they study early mitotic events and their roles in KV morphogenesis. Through genetic and chemical perturbations and laser ablations, the authors find that fgf signaling promotes mitosis in a small population of anteriorly positioned KV cells at early stages. Surprisingly, these early mitotic events of just a few cells play an outsized role in morphogenesis and lumen formation relative to those of later stages. Finally, the authors find that early dividing KV cells have a unique function in cell rearrangements that are necessary for growth and morphogenesis of the organ. Overall, the ablation experiments are well designed, the main conclusions are supported by the data presented, and the findings will be of interest to developmental biologists.

SUGGESTIONS TO AUTHORS

Essential revisions:

1) Ablation of early mitotics
a. Have the authors performed experiments in which only a small proportion of early mitotics are ablated rather than all of them (for example, ablating only 1 or 2 of the first 4 mitotics), and does this lead to an intermediate lumen phenotype? This control would help to more convincingly show the morphogenetic phenotypes are not an artifact of KV ablation at the early stage. Alternatively, this concern could be addressed if the authors have performed a control experiment of ablating non-mitotic cells at the same exact stage of the early mitotic ablations (during the first 4 divisions).
To address the reviewer’s concerns, we performed a control experiment ablating 6-8 non-mitotic cells, with 3-4 ablations before the KV reached 20 cells and the rest after. Lumen formation remained relatively unaffected (Fig. 7E, 7F). These same controls were performed in Fig. 6B and we found no significant defects in circularity. These non-mitotic ablation controls rule out both early-stage and later stage non-mitotic cell ablation as a cause of KV developmental defects. Additionally, we performed ablating a single mitotic cell before the KV reached 20 cells. Here we found that KVs had normal development (now included in Fig. 7D, 7E). These findings suggest that while early mitotic events are critical for lumen formation, the loss of an individual mitotic event can likely be compensated by others at this stage.

2) There appears to be an error of the x axis of Fig. 3B.
Fixed to min.

Additional minor comments:

3) Pericentrin mutants
a. Given that a significant proportion of pcnt mutants die during the experimental window (by 12 hpf), can the authors clarify whether the analyses in Fig.1 E-H performed on mutants that did not show widespread necrosis?
We thank the reviewer for this important point. To clarify, the analyses presented in Fig. 1E-H were conducted exclusively on *pcnt* mutants that did not exhibit widespread necrosis. Importantly, this

© 2025. Published by The Company of Biologists under the terms of the Creative Commons Attribution License (https://creativecommons.org/licenses/by/4.0/).

subset of mutants is viable and able to develop fully into adulthood, as further demonstrated by our heart development studies. In practice what we observe with embryos entering necrosis or death is that in brightfield the turbidity of the tissue increases, these embryos were not used for KV analysis and were included in our survival curves. Only embryos that exhibited a healthy state and transparent structures were sorted for fixing and processing, dead or dying embryos were disposed of and counted for in Figure 1A and 1B.

b. To show that loss of pericentrin function in KV is responsible for the phenotypes shown in Fig.1 C-H, a rescue experiment would be needed, for example by expressing sox17:pcnt in mutants. I don't consider this essential because the ablation experiments generally support the conclusion, but this should be noted.
We have edited the text to note this.

4) Relationship of early and late mitotic events to trafficking of CFTR
a. It would be helpful to briefly discuss their results in the context of their prior work on mitosis in KV. Although this is alluded to in the discussion section, can the authors comment on how early versus late mitotic events might relate to the apical positioning of Rab11 and CFTR that is needed for lumen expansion?
We have added a discussion of these points (refer to highlighted sections in discussion).

5) Fgf
a. It would be helpful for the authors to cite a paper showing fgf signaling activity in DFCs and in KV (Molina et al., 2007. BMC Dev Biol).
We have added Molina et al.

Second decision letter

MS ID#: dev.204687R1

MS TITLE: Specific Mitotic Events Drive Left-Right Organizer Development

AUTHORS: Yan Wu, Yiling Lan, Favour Ononiwu, Abigail Poole, Kirsten Rasmussen, Jonah Da Silva, Abdalla Wael Shamil, Li-En Jao and Heidi Hehnly

Dear Dr Hehnly,

I am happy to tell you that your manuscript has been accepted for publication in Development, pending our standard publication integrity checks.

Reviewer 1

This manuscript investigates early stages of Zebrafish development in the context of the KV and the role of mitotic cell division. This perspective is rarely investigated as Zebrafish is more often used to study later stages of development. However, early development is extremely important and this lab has established an impressive body of work in this timeline of dvelopment, especially with respect to mitotic spindle positioning and centrosome positioning. I am strongly supportive of publication of this manuscript.

Comments for the author

The authors have more than satisfied all my initial critiques of this first draft of the manuscript.

Reviewer 2

© 2025. Published by The Company of Biologists under the terms of the Creative Commons Attribution License (https://creativecommons.org/licenses/by/4.0/).

The authors have addressed my concerns by improving the ablation experiments by adding additional controls. I recommend the manuscript for publication and congratulate the authors on their interesting study.

© 2025. Published by The Company of Biologists under the terms of the Creative Commons Attribution License (https://creativecommons.org/licenses/by/4.0/).
